# Coupling of nanostraws with diverse physicochemical perforation strategies for intracellular DNA delivery

**DOI:** 10.1186/s12951-024-02392-w

**Published:** 2024-03-26

**Authors:** Juan Jiang, Jing Liu, Xinmin Liu, Xingyuan Xu, Zhengjie Liu, Shuang Huang, Xinshuo Huang, Chuanjie Yao, Xiafeng Wang, Yixin Chen, Hui-jiuan Chen, Ji Wang, Xi Xie

**Affiliations:** 1 Institute of Precision Medicine, The First Affiliated Hospital, Sun Yat-sen University, Guangzhou, 510080 Republic of China; 2 State Key Laboratory of Optoelectronic Materials and Technologies, Guangdong Province Key Laboratory of Display Material and Technology, School of Electronics and Information Technology, Sun Yat-Sen University, Guangzhou, 510006 Republic of China; 3 Sun Yat-sen University Zhongshan School of Medicine, Guangzhou, 510080 Republic of China

**Keywords:** Nanostraw, Intracellular delivery, Physical field coupling, Nano-electroporation, Cell penetration

## Abstract

Effective intracellular DNA transfection is imperative for cell-based therapy and gene therapy. Conventional gene transfection methods, including biochemical carriers, physical electroporation and microinjection, face challenges such as cell type dependency, low efficiency, safety concerns, and technical complexity. Nanoneedle arrays have emerged as a promising avenue for improving cellular nucleic acid delivery through direct penetration of the cell membrane, bypassing endocytosis and endosome escape processes. Nanostraws (NS), characterized by their hollow tubular structure, offer the advantage of flexible solution delivery compared to solid nanoneedles. However, NS struggle to stably self-penetrate the cell membrane, resulting in limited delivery efficiency. Coupling with extra physiochemical perforation strategies is a viable approach to improve their performance. This study systematically compared the efficiency of NS coupled with polyethylenimine (PEI) chemical modification, mechanical force, photothermal effect, and electric field on cell membrane perforation and DNA transfection. The results indicate that coupling NS with PEI modification, mechanical force, photothermal effects provide limited enhancement effects. In contrast, NS-electric field coupling significantly improves intracellular DNA transfection efficiency. This work demonstrates that NS serve as a versatile platform capable of integrating various physicochemical strategies, while electric field coupling stands out as a form worthy of primary consideration for efficient DNA transfection.

**Supplementary Information:**

The online version contains supplementary material available at 10.1186/s12951-024-02392-w.

## Introduction

Intracellular drug delivery stands as an indispensable prerequisite propelling the progress of cell-based therapy and gene therapy technologies. This holds particularly true for the immensely promising nucleic acid drug molecules, including DNAs and RNAs [1, 2]. However, the cell membrane’s negative charge, due to its phospholipid bilayer structure and surface protein modifications, presents a significant barrier to negatively charged nucleic acid molecules. Current cell transfection methods help overcome this barrier by facilitating the movement of nucleic acids into the cytoplasm and eventually to the nucleus for transcription. Common transfection methods use chemical carriers like liposomes and positively charged polymers to encapsulate nucleic acids, neutralizing their charge and enhancing interaction with the cell membrane [3, 4]. However, these methods have limitations including potential risks associated with carrier residues and the reliance on endosomal escape post cellular endocytosis [5]. Biocarriers like membrane-penetrating peptides and viral vectors pose safety concerns, limiting their clinical use [6, 7]. In contrast, physical force-based intracellular delivery methods offer a direct approach without introducing foreign agents. These methods include mechanical forces [8, 9], laser pulses [10, 11], and electricity [12], which facilitate the entry of nucleic acid molecules into cells. Established methods like electroporation and microinjection have successfully secured commercial footholds [13–15], but they have drawbacks such as cytotoxicity due to the ultra-high voltages (~ 200 V) for bulk electroporation [16, 17], and limitations in manipulating multiple cells simultaneously due to the intricate and time-consuming nature of microinjection [18].

Nanoneedle arrays, characterized by their nanometer-scale diameters, have showcased significant strides in the advancement of drug delivery into cells [19–21]. These arrays, fabricated through techniques like micromachining or chemical growth, offer a stark departure from conventional microinjection methods, boasting diameters spanning from 100 to 500 nm. When cells are cultured on these arrays, their membranes embrace the nanoneedles, creating a tight interface [22, 23]. The high height-to-diameter ratio and finely honed structure of nanoneedles hold the potential to disrupt cell membrane during growth, facilitating the diffusion of molecules absorbed onto their surface or dispersed within a solution [24, 25]. The pivotal advantage of this delivery methodology lies in the direct access it provides for drug molecules into the cell cytoplasm, bypassing the need for cellular endocytosis and endosomal escape [26–28]. Furthermore, the feasible of crafting expansive arrays of nanoneedles paves the way for simultaneous intracellular delivery to a multitude of cells. Additionally, owing to the significantly smaller diameter of nanoneedles compared to typical cells (~ 10 μm), the disruption area incurred by nanoneedle penetration is minimized.

Despite these benefits, a growing body of research emphasizes the challenges of relying solely on gravitational or adhesive forces for membrane perforation [29]. Even in occasional instances of successful membrane perforation, there is considerable variance in final delivery efficiency. To amplify the effectiveness of nanoneedle penetration through cell membranes, the application of external physical forces or chemical modifications has emerged as a promising strategy. These mechanisms entail the integration of physical fields, such as mechanical forces, laser irradiation, and electric field, or chemical modifications, with the nanoneedles to bolster penetration efficiency. For example, Wang et al. harnessed centrifugal force and nanoneedle arrays to deform cell membranes, leading to a remarkable eight-fold enhancement in the transfection efficiency of primary cells with plasmid DNA [30]. Laser irradiation of metal nanoneedles can generate photothermal heat, breaking cell membranes, while high-velocity nanoshockwaves can also perforate membranes [31]. Liu et al. extended this exploration by synergizing nanoneedles with electrical stimulation, achieving successful delivery of fluorescent molecules through cell membranes [32]. Furthermore, the application of chemical modifications or auxiliary agents to nanoneedles hold the potential to augment membrane perforation and overall transfection efficiency [29, 33]. In summation, the application of external physical forces or chemical modifications offers a promising avenue for augmenting the capability of nanoneedle structures to traverse the cell membrane’s phospholipid bilayer, thereby promoting intracellular delivery. However, it is crucial to acknowledge that conventional solid nanoneedles indeed have limitations in active drug delivery and in precisely controlling the timing or repeating of the drug delivery process, whether the molecules are adsorbed on the surface or dispersed within the culture medium.

**Fig. 1:**
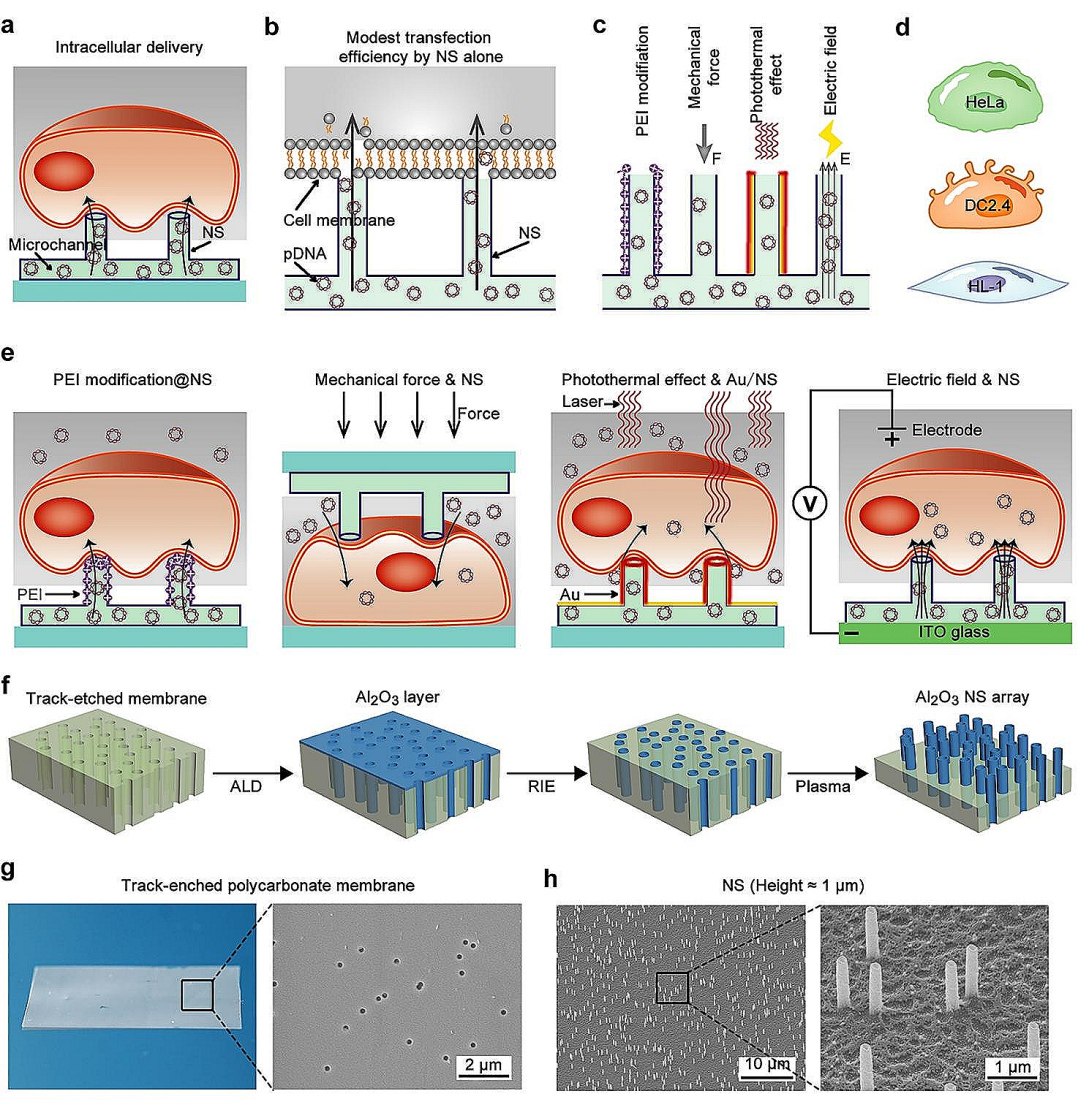
Schematic representation of NS coupling with various physicochemical perforation for intracellular DNA delivery. (**a**) Schematic representation of NS integrated with a microfluidic design tailored for intracellular delivery. (**b**) Transfection model illustrating NS alone-mediated modest transfection without the application of external forces. (**c**) Schematic representation of NS coupling with four distinct physicochemical perforation methods for transfection, including cationic ionic polymer PEI modification, mechanical force, photothermal effect, and electric field. (**d**) Illustration of three distinct cell types used to test transfection efficiency: epithelial-like cells (HeLa), immune cells (DC2.4), and myocardial cells (HL-1). (**e**) Four customized settings designed for application in one of the four physicochemical perforation coupling scenarios involving NS. (**f**) Schematic demonstrating the fabrication procedure for the array of hollow Al_2_O_3_ NS. (**g**) Photograph (left) and SEM image (right) of the TPM. (**h**) SEM image of NS with a height of 1 μm, with an enlarged view provided on the right

Diverging from solid nanoneedles, nanostraws (NS) represent nanoneedle-like structures characterized by hollow tubes, conferring upon them the unique capability of facilitating the flexible delivery of solutions [33–35]. Consequently, they possess a slew of advantages over the solid nanoneedles in the realm of intracellular drug delivery. Employing a microfluidic design, the lower solution storage chamber and the upper cell culture cavity are constructed and integrated with NS to create a device tailored for cellular delivery [36] **(**Fig. 1a**)**. This specialized set not only facilitates the precise adjustment of drug concentration and composition but also enables flexible control over the timing and repetitive intracellular administration aligned with specific requirements. However, analogous to the limitations encountered with solid nano-needles, the cellular delivery efficiency remains quite modest through NS, relying solely on the cell’s inherent gravity and adhesion **(**Fig. 1b**)**. To bolster delivery efficiency, it becomes imperative to leverage a combination of external physical fields (electric field, mechanical force, and laser) and chemical modification integrated with NS. Nevertheless, a notable gap prevails in comprehensive and comparative investigations assessing the potential of these physicochemical methods in tandem with NS to facilitate the perforation of the cell membrane, thereby augmenting the efficiency of cellular delivery.

In this study, we couple the hollow alumina (Al_2_O_3_) NS with four distinct physicochemical perforation methods, including cationic polymer PEI modification, mechanical force, photothermal effect, and electric field **(**Fig. 1c**)**. These combinations are employed in intracellular DNA transfection with the objective of enhancing transfection efficiency. Our investigations span across three representative cell lines, including epithelial cell line HeLa, immune cell line DC2.4, and cardiac cell line HL-1 **(**Fig. 1d**)**. NS is incorporated into four customized settings, each specifically designed for application in one of the four physicochemical perforation coupling scenarios involving NS **(**Fig. 1e**)**. Within the ambit of this study, we conduct a systematic exploration into the synergistic effects of coupling NS with various physicochemical perforation methods on DNA transfection efficiency and cellular safety. For each coupling technique, we examine an array of applied intensity conditions to optimize transfection efficiency while ensuring minimal impact on cell viability. Our results show that the coupling strategies involving NS with PEI modification, mechanical force, photothermal effects, exhibit a modest impact on enhancing DNA transfection. Excessive mechanical force and photothermal effects exert a significant toll on cell viability. Conversely, electric field-coupled NS remarkably prompt DNA transfection, showcasing substantial improvements across three distinct cell lines. This emphasizes the versatility of NS as a platform proficient in integrating various physicochemical perforation methods. Proper selection of coupling strategies and the optimization of conditions are crucial factors for promoting efficient DNA transfection. This study establishes a reliable platform for testing and developing high-throughput, secure, and efficient DNA transfection methodologies, contributing to the progression of cell therapy and gene therapy technologies.

## Results and discussion

### Nanostraw fabrication, characterization and cell culturing

In this study, track-etched polycarbonate membranes (TPM) serve as templates to fabricate NS. The 25 μm-thick membrane features evenly distributed vertical nanochannels spanning from the bottom to the top surface, which act as molds for shaping NS. Critical parameters of the NS, such as pore diameter, length, and distribution density on the substrate, can significantly impact cell transfection efficiency. Given the typical cell spreading length on substrates falls within the order of 10 μm, a TPM boasting nanochannels with 200 nm diameter is chosen as the ideal template for NS fabrication. The diameter represents 2% of the cell spreading length, thus avoiding potential cellular damage caused by large-area perforation. Additionally, the distribution density of NS emerges as a pivotal factor influencing the safety and intracellular delivery efficiency. Based on preliminary experimental results [16], a TPM with a distribution density of 2 × 10^7^ nanochannels/cm² is selected as the template, resulting in an average spacing of 2.2 μm between each pair of NS. Following a well-established procedure to fabricate the NS [37], the initial step involves depositing a ~ 20 nm thick layer of Al_2_O_3_ onto the surface of the TPM and the inner walls of all nanochannels through atomic layer deposition (ALD) **(**Fig. 1f**)**. Subsequently, reactive ion etching (RIE) is employed to completely remove the Al_2_O_3_ layer from the surface of TPM, exposing the underlying polycarbonate material. The Al_2_O_3_ NS are then formed after additional oxygen plasma etching. The depth of the TPM removed via oxygen plasma etching is precisely controlled to yield NS of desired height. The height of NS is another crucial element that influence the cell growth and delivery efficiency. Previous study has demonstrated that NS measuring around 1 μm in height efficiently enhance cellular drug delivery without significant cytotoxicity [38, 39]. In this study, precise control of the etching time was implemented to attain NS measuring 1 μm in length, forming an array of NS. Scanning electron microscopy (SEM) characterization shows the uniform distribution of nanochannels with a diameter of 200 nm on the surface of the TPM (Fig. 1g, Fig. S1a). Following the aforementioned micro-nano fabrication process, an array of Al_2_O_3_ NS with a height of 1 μm is effectively cultivated within the nanochannels, presenting a consistent vertical tubular structure adorned with smooth orifices at the tip (Fig. 1h, Figure S1b).

To facilitate cell cultivation and the coupling with various physicochemical perforation methods, a cell culture device is constructed for NS-based delivery by integrating NS with cell culture chamber and microchannel. The NS is positioned above the microfluidic groove, while the cell culture chamber is situated above them. And the microfluidic groove is stuck to a glass substrate. Additionally, there are two throughout sample injection holes besides the cell culture chamber, which connect with the lower microchannel. The lower microchannel layer and upper cell culture chamber layer are fabricated using polydimethylsiloxane (PDMS) cross-linking through molding. The TPM, hosting the NS array, function as a central layer between the microchannel and the cell culture chamber, forming a sandwich structure (Figure S2). As a result, this allows for dynamic control over the concentration of DNA molecules within the microchannel solution, providing flexibility in regulating the applied DNA concentration to the cells.

**Fig. 2:**
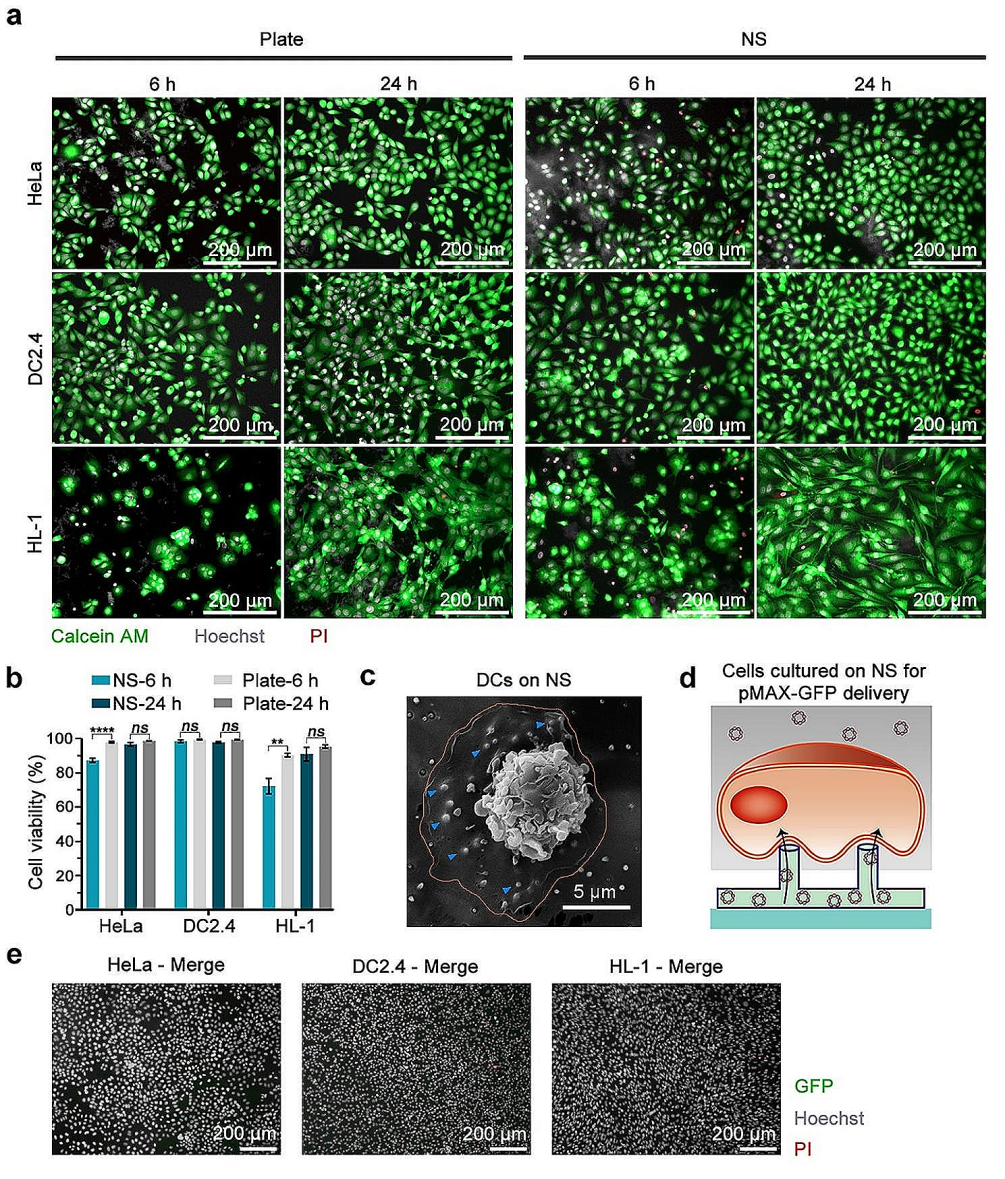
Cells cultured on NS and pMAX-GFP transfection through NS without external forces. (**a**) Fluorescent microscopy images displaying the growth and spreading of HeLa, DC2.4, and HL-1 cells on the conventional Plate and NS over a 6 or 24-h period after seeding. Calcein AM (green), Hoechst (gray), and PI (red) are used for cell labeling. The upper panel shows HeLa cells, the middle panel represents DC2.4 cells, and the lower panel displays HL-1 cells. The left two rows show cells cultured on a conventional cell culture plate (Plate), and the right two rows represent cells cultured on the NS. (**b**) Quantification of cell viability after 6 and 24 h of culture on the NS. Mean ± SEM, *n* = 3 regions, Two-way ANOVA. (**c**) SEM image of DCs on the NS after 24 h cultivation. The orange line illustrates the border of the cell, and the blue arrows indicate the NS beneath DCs. (**d**) Schematic illustration of cells cultured on the NS for pMAX-GFP delivery without external force. (**e**) Fluorescent microscopy images of HeLa, DC2.4, and HL-1 cells expressing GFP at 24 h after NS-mediated delivery of pMAX-GFP without external forces, respectively. The images display the merged channel of GFP (green), Hoechst (gray), and PI (red). The scale bars in all images are 200 μm

Before cell culturing, the surface of the NS is initially coated with fibronectin (Fn) to enhance cell adhesion and spreading upon attachment. HeLa, DC2.4, and HL-1 cells are then cultured in the cell culture chamber within the device, and their biocompatibility is assessed by cell condition, involving the morphology and viability. Simultaneously, cells with the same concentrations are seeded in a conventional 96-well plate, serving as the control, to facilitate a comparison of cell spreading over time on two distinct substrates. After 6 and 24 h of cell culture, the cells are stained with Calcein AM, Hoechst, and propidium iodide (PI) to label the cytoplasm of live cells, cell nucleus, and dead cells, respectively, followed by observation under fluorescence microscopy. The microscopy imaging illustrates that the HeLa and DC2.4 cells are growing well on both substrates and exhibits a gradual spreading state after 6 h and full spreading after 24 h. HL-1 cells adhere to the NS after 6 h but do not fully spread until 24 h, displaying the typical spindle cardiomyocyte morphology (Fig. 2a, Fig. S3). Compared to conventional cell culture plates, NS culture conditions exhibit minimal impact on cell growth characteristics, as evidenced by the similar morphology observed between the two distinct conditions. Additionally, the viability of HeLa, DC2.4, and HL-1 cells on the NS after 24 h of culture exceeds 90% (Fig. 2b). However, at 6 h, the viability of HeLa and HL-1 cells on the NS is slightly lower than that on the culture plates, indicating that some cells have not yet adapted to the NS culture environment at the initial stage. Overall, cells are able to grow healthily on the NS, and the vertical nanostructure does not exhibit significant cytotoxicity, demonstrating the good biocompatibility of the NS.

To directly visualize the interaction between cells and the NS, DCs are cultured on the NS for 24 h before imaging with SEM. Our observations reveal that DCs have stabilized and displayed complete adhesion and spreading on the NS. The NS is distinctly visible beneath the DCs, indicating the formation of a robust biophysical interface between them (Fig. 2c). However, there is potential of cell membrane rupture and perforation during the growth on NS. Therefore, we first conduct a validation to demonstrate whether the cell membrane is able to be pierced by the NS alone for plasmid DNA delivery. Once the cell membrane is pierced by the NS, the unique hollow tubular architecture, extending vertically from the lower surface of the polycarbonate membrane to the top, facilitates the diffusion of the solution from the lower microchannel into the upper section of the NS. Here, the green fluorescence protein (GFP) encoded plasmid DNA (pMAX-GFP) is engaged as a nucleic acid molecular tool to characterize the intracellular delivery efficiency through the NS. The pMAX-GFP solution is introduced into the cell culture medium and the lower microchannel with a final concentration of 0.1 µg/µl, and the cells undergo an additional 24 h of cultivation (Fig. 2d). Then cells are stained with Hoechst and PI staining for comprehensive cell counting and evaluating the number of dead cells. As it shows in the microscopy imaging, almost no any expression of GFP from all the three types of cells after 24 h post pMAX-GFP introduction, suggesting unsuccessful membrane perforation (Fig. 2e). These results validate that either the gravitational forces of the cell or the adhesive forces between cells and the NS are insufficient to enable the penetration of the cell membrane, emphasizing the necessity for additional external energy or chemical modifications to augment the cell membrane penetration efficiency of NS.

Subsequently, we employ distinct coupling approaches for NS, involving cationic polymer PEI modification, mechanical force, photothermal effects, and an electric field. Each of these diverse coupling strategies is applied individually for DNA transfection into cells, and we systematically compare their relative efficacy in enhancing transfection efficiency.

### Nanostraws coupled with PEI modification for cellular DNA transfection

**Fig. 3:**
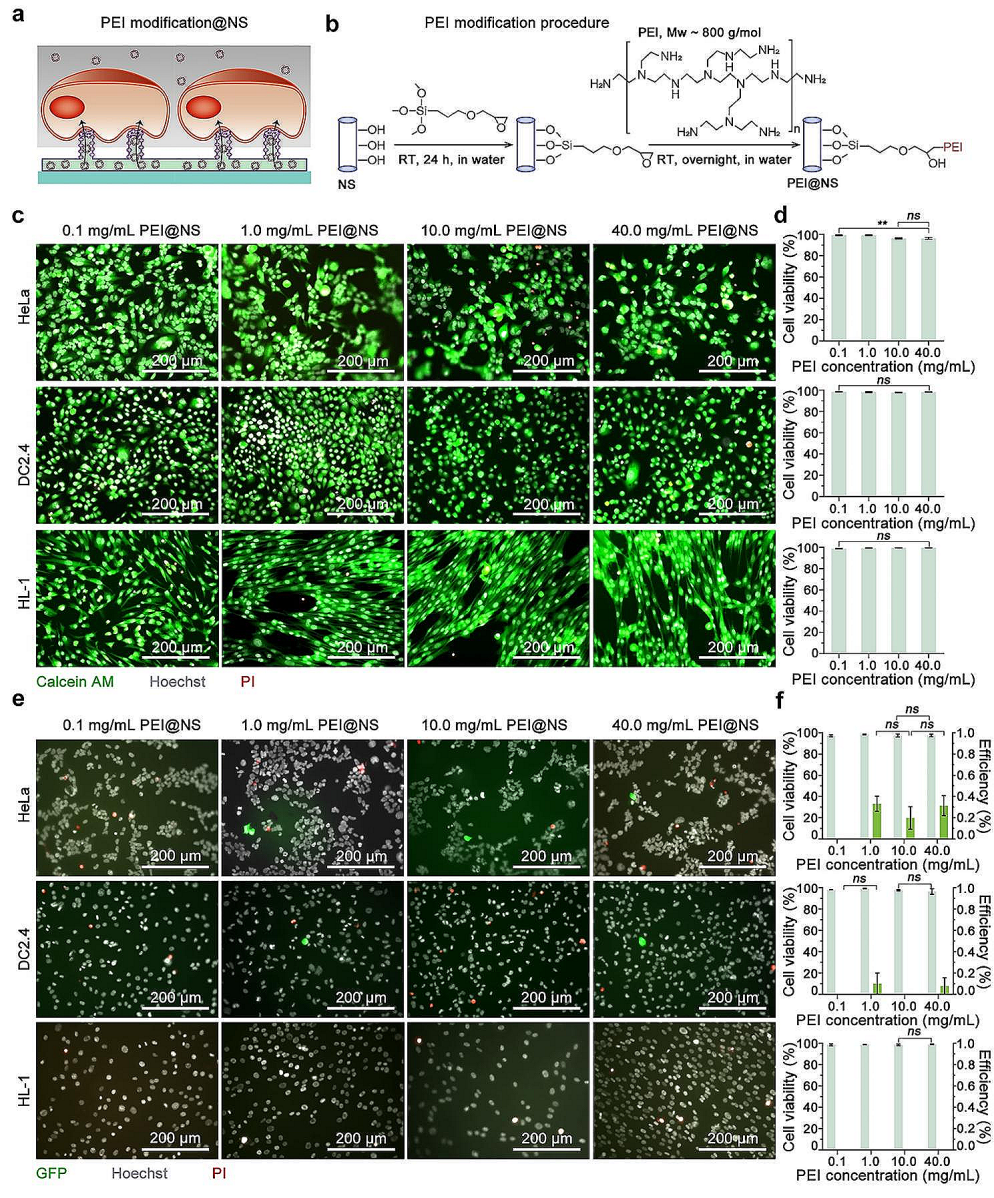
NS coupled with PEI modification for cellular DNA transfection. (**a**) Schematic illustration outlining the transfection by NS coupled with PEI modification (PEI modification@NS). (**b**) Depiction of the PEI modification procedure of the NS. (**c**) Fluorescent microscopy images illustrating the cell condition on the PEI@NS context over a 24-h period after initial cell seeding. Merged signals from Calcein AM (green), Hoechst (gray), and PI (red) are presented. The upper panel represents HeLa cells, the middle panel shows DC2.4 cells, and the lower panel features HL-1 cells. Cell condition on PEI@NS modified with various concentrations of PEI (0.1, 1.0, 10.0, and 40.0 mg/mL) solution are arranged from left to right. (**d**) Quantification of cell viability on the PEI@NS based on microscopy images. Mean ± SEM, *n* = 3, One-way ANOVA. (**e**) Fluorescent microscopy images showcasing cells expressing GFP following transfection through PEI@NS. These images display the merged signals of GFP, Hoechst, and PI, and the rows correspond to those shown in panel (**c**). (**f**) Quantification of transfection efficiency and cell viability after pMAX-GFP transfection mediated by PEI@NS, based on the microscopic images. Mean ± SEM, *n* = 3, Two-way ANOVA. Scale bars in all images are 200 μm

We initiate an exploration into the potential of NS in intracellular DNA transfection following chemical modification. We investigate PEI, a prominent cationic polymer, to assess its capacity to modify NS, aiming to evaluate its potential in penetrating cell membranes and facilitating DNA transfection (Fig. 3a). Cationic polymers are commonly employed in nanoparticle modification to enhance endocytosis, facilitating substance delivery into cells. Since cell membranes typically carry a negative charge, the PEI modification proves advantageous for enhancing the binding force between the cell membrane and NS. This robust binding force potentially induces stability changes in the double-layer phospholipid structure of the cell membrane, facilitating the perforation of the cell membrane. Specifically, the PEI modification procedure involves chemically bonding hydroxyl groups on the surface of Al_2_O_3_ NS with a silane coupling agent. Subsequently, solutions of PEI at concentrations of 0.1, 1.0, 10.0, and 40.0 mg/mL are introduced, followed by overnight incubation for modification reaction (Fig. 3b). Additionally, to corroborate the successful modification of PEI onto NS, a fluorescent gorup, Cy5, is conjugated to PEI subsequent to its modification onto the NS, yielding PEI-Cy5@NS (Figure S4a). Following this, PEI-Cy5@NS undergo fluorescence microscopy imaging. The unmodified NS and NS modified with a silane coupling agent (Silane@NS), utilized as controls, exhibit a failure to bind with Cy5. However, the observable fluorescence emitted by Cy5 on the PEI-Cy5@NS, which becomes more pronounced with higher concentrations of PEI modification, confirms the successful PEI modification (Figure S4b).

The PEI-modified NS (PEI@NS) are integrated with microchannel into cell culture devices and HeLa, DC2.4, and HL-1 cells are subsequently seeded on PEI@NS for cell viability detection to assess the influence of PEI modification. Cells are stained with Calcein AM, PI, and Hoechst after a 24-h culture period on PEI@NS, followed by observation through a microscope. Robust cell adhesion is observed, with consistently high viability rates (> 95%) for all cell types, indicating that PEI modification does not compromise cell health, albeit with a very slight reduction in viability observed in HeLa cells (Fig. 3c and d, Figure S5a). We proceed to test the feasibility of PEI@NS for DNA transfection into cells. A solution containing pMAX-GFP is applied to the cell culture medium and the microchannel below the PEI@NS with a concentration of 0.1 µg/µL. This ensures a sufficient presence of plasmid DNA around the cells, preventing diffusion difficulties through the NS that could potentially affect the final transfection efficiency. After the introduction of pMAX-GFP, cells are cultured for an additional 24 h, followed by PI and Hoechst staining. Transfection efficiency is calculated as the ratio of cells expressing GFP to the total cell count. Fluorescence microscopy observations reveal that, after modifying NS with 1.0, 10.0, and 40.0 mg/mL of PEI, only sporadic HeLa and DC2.4 cells are successfully transfected, with GFP expression efficiency below 1% (Fig. 3e and f, Figure S5b). There is almost no expression of GFP observed in HL-1 cells. Although the cell viability after transfection validate that PEI modification indeed does not induce cytotoxicity or compromise cell activity, it fails to enhance the pMAX-GFP transfection efficiency in HeLa, HL-1, and DC2.4 cells significantly. Several factors may account for this compromised efficiency. On one hand, PEI molecules are exclusively modified on the surface of Al_2_O_3_ NS and not on the TPM. The limited surface area of the NS results in a relatively sparse modification of PEI molecules, leading to restricted cell membrane permeability. On the other hand, the closely adherent cell membranes to the substrate present a challenge for the entry of plasmid DNA molecules, even following cell membrane penetration through coaction with PEI. To achieve both cell membrane penetration and efficient plasmid DNA delivery in this system, a potential strategy could involve introducing a solution containing PEI molecules and plasmid DNA into the microchannel. This approach may facilitate the entry of plasmid DNA molecules following cell membrane penetration at the tips of NS. Nevertheless, a critical consideration is the low diffusion efficiency of plasmid DNA molecules in aqueous solutions, posing limitations on the efficacy of free diffusion into cells.

### Nanostraws coupled with mechanical force applied to cellular DNA transfection

**Fig. 4:**
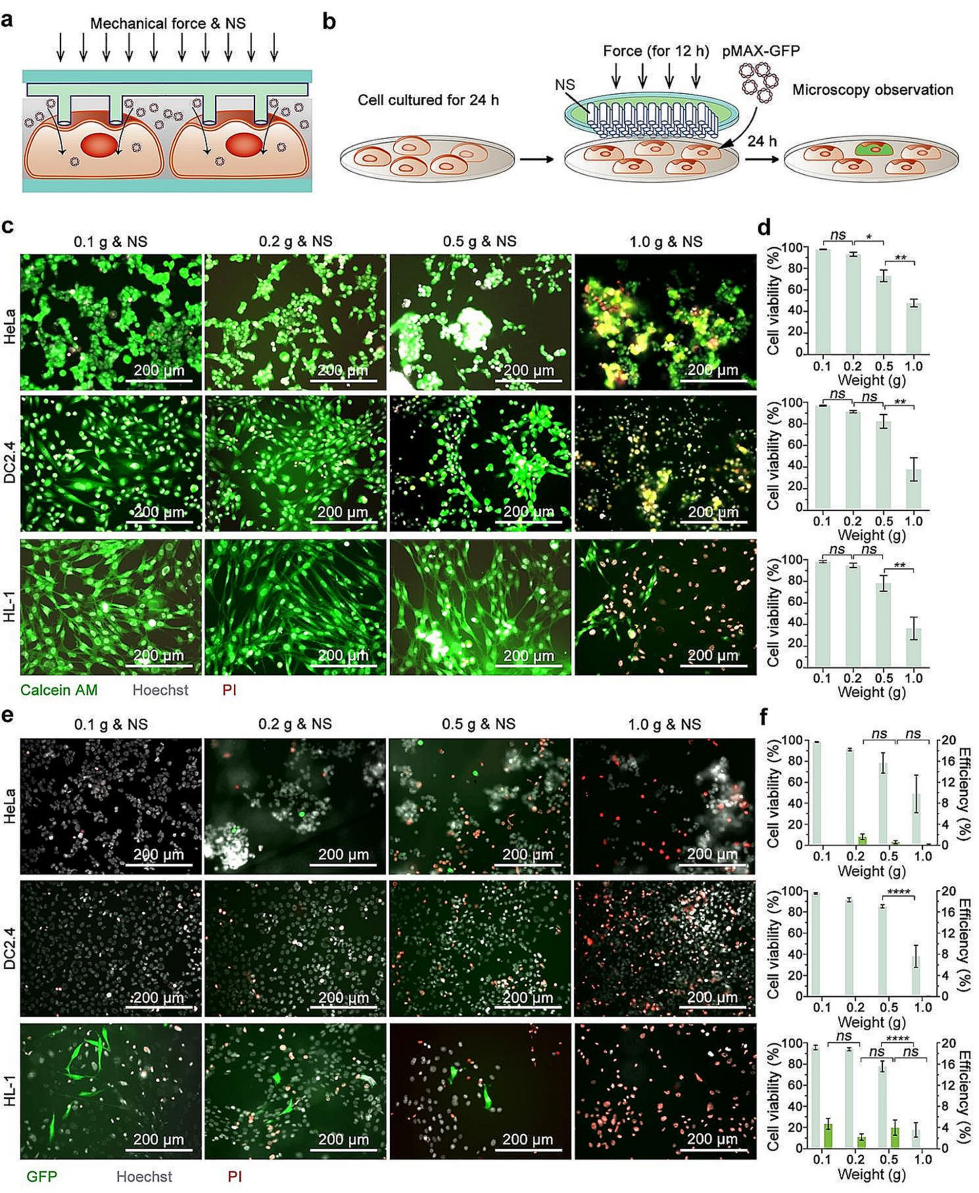
NS coupled with mechanical force applied to cellular DNA transfection. (**a**) Schematic illustrating the transfection by NS coupled with mechanical force (Mechanical force & NS). (**b**) Depiction of the procedure involved in applying mechanical forces to the NS to facilitate transfection efficiency. (**c**) Fluorescent microscopy images showing the cell condition to mechanical forces applied to the NS over a 12-h period. The images display the merged signals from Calcein AM (green), Hoechst (gray), and PI (red). The upper panel represents HeLa cells, the middle panel shows DC2.4 cells, and the lower panel features HL-1 cells. Results of cell condition after different weights (0.1, 0.2, 0.5, and 1.0 g) application are presented in rows from left to right. (**d**) Assessment of cell viability under different mechanical force conditions based on microscopic images. Mean ± SEM, *n* = 3, One-way ANOVA. (**e**) Fluorescent microscopy images displaying cells expressing GFP following delivery via NS coupled with mechanical force. These images represent the merged signals of GFP, Hoechst, and PI, with the rows corresponding to those in panel (**c**). (**f**) Evaluation of transfection efficiency and cell viability after pMAX-GFP transfection mediated by NS coupled with mechanical force, based on the microscopic images. Mean ± SEM, *n* = 3, Two-way ANOVA. Scale bars in all images are 200 μm

Due to the slender structure of the NS, we therefore consider to apply external mechanical force to facilitate NS in penetrating the cell membrane, investigating the feasibility of DNA transfection (Fig. 4a). The application of external mechanical force is beneficial as it enhances pressure exerted by NS on the cell membrane, promoting the attainment of the energy barrier necessary for breaching the cell membrane. Initially, HeLa, DC2.4, and HL-1 cells are cultured in standard 48-well culture plate. All cell types undergo a 24-h culture period to facilitate adhesion to the substrate and achieve better spreading, ensuring a more uniform force application on the cells. Subsequently, the TPM with NS is affixed to a coverslip, then inversely positioned onto the cell surface, allowing the NS to establish direct vertical contact with the cells. Additional weights, ranging from 0.1 to 1.0 g including the weight of the coverslip, are applied to the back of the TPM, subjecting cells to four different forces for a duration of 12 h (Fig. 4b). Considering the potential for external force to induce alterations in cell morphology or behavior, along with the risk of mechanical force causing cell membrane rupture and subsequent cell death, our initial step involves assessing the cell condition. Following the removal of NS and mechanical force, cells are stained with Calcein AM, PI, and Hoechst for subsequent microscopy observation. The results reveal that coupling NS with 0.1 and 0.2 g weights has no significant impact on cell viability or morphology, maintaining cell viability of HeLa, DC2.4, and HL-1 consistently above 90%. However, under the weight of 0.5 g, the cell viability is notably affected. Specifically, the viability of HeLa cells is reduced to 73.05%, DC2.4 cells exhibit viability of 82.33%, and HL-1 cells show viability of 78.15%. Moreover, HeLa cells tend to form clusters when subjected to an external weight of 0.5 g, indicating a gradual impact on cell status with increased pressure. Notably, when exposed to NS combined with a weight of 1.0 g, HeLa cell viability decreases to 47.80%, DC2.4 cells to 37.96%, and HL-1 cells to 36.51% (Fig. 4c and d, Figure S6a), suggesting that excessive pressure significantly impairs cell viability.

Having gained comprehensive insights into the impact of coupling NS with various external mechanical forces on cellular activity, we leverage this understanding to enhance cellular DNA transfection. In this process, a solution containing pMAX-GFP is introduced to the cell culture medium at a final concentration of 0.1 µg/µl. As previous, cells are subjected to varying degrees of external force for a duration of 12 h before removing the external weight and the NS. The cells are then cultured for an additional 24 h, allowing sufficient time for GFP expression (Fig. 4b). Post-incubation, the cells are subjected to PI and Hoechst staining for fluorescence characterization. The results show that, the external force provided by the weight of 0.1 g does not lead to transfection in HeLa cells. When subjected to weights of 0.2, 0.5, and 1.0 g coupled with NS, only a limited number of HeLa cells successfully undergo transfection, albeit with a GFP expression efficiency below 2%. The impaired cell activity with 48.91% under the highest degree of the external force supplied by a weight of 1.0 g. Notably, DC2.4 cells exhibit no transfection under any of the four conditions, while the cell viability significantly reduces to 38.21% in the scenario of NS coupling a weight of 1.0 g. Among HL-1 cells, GFP expression is observed under the weights of 0.1, 0.2, and 0.5 g, yielding transfection efficiencies of 4.71%, 2.22%, and 4.00%, respectively, with no significant variance (Fig. 4e and f, Figure S6b). However, the transfection effects are notably inhomogeneous, with several local areas failing to exhibit transfection results. The higher weight of 1.0 g results in declined viability to 17.85%, with no contribution to pMAX-GFP transfection. In comparison to HeLa and DC2.4 cells, HL-1 cells demonstrate a relatively higher efficiency of successful transfection mediated by NS coupling with mechanical forces. This could be attributed to the well-spreading nature of HL-1 cells, increasing the likelihood of cell membrane perforation under mechanical force. In contrast, DC2.4 cells have no benefits from NS coupling external forces strategy in the aspect of cellular DNA transfection. This is likely due to the softness of DC2.4 cells and their highly fluid cell membrane, which hinders membrane penetration by NS assisted with mechanical force. Despite this, excessive external mechanical force still results in a significant decrease in cell viability, highlights that the damage caused by mechanical force is irreversible.

These outcomes indicate that coupling with external mechanical force does not effectively enhance the ability of NS to transfect DNA into cells. The limited success of this coupling strategy may be attributed to multiple complicated reasons. Although the NS features a slender nanoneedle-like structure, the top smooth orifice, lacking a sharp needle-tip effect, may not facilitate the penetration of cell membranes under mechanical forces. Furthermore, the uneven surface of cell membranes and the relatively limited length of NS, pose a challenge in ensuring a uniform distribution of force on the cell membrane surface, consequently influencing their cellular status and transfection efficiency. Of note, it is difficult to precisely manipulate the application of mechanical force during the transfection process, which may also lead to the heterogeneous transfection efficiency.

### Nanostraws coupled with photothermal effect applied to cellular DNA transfection

**Fig. 5:**
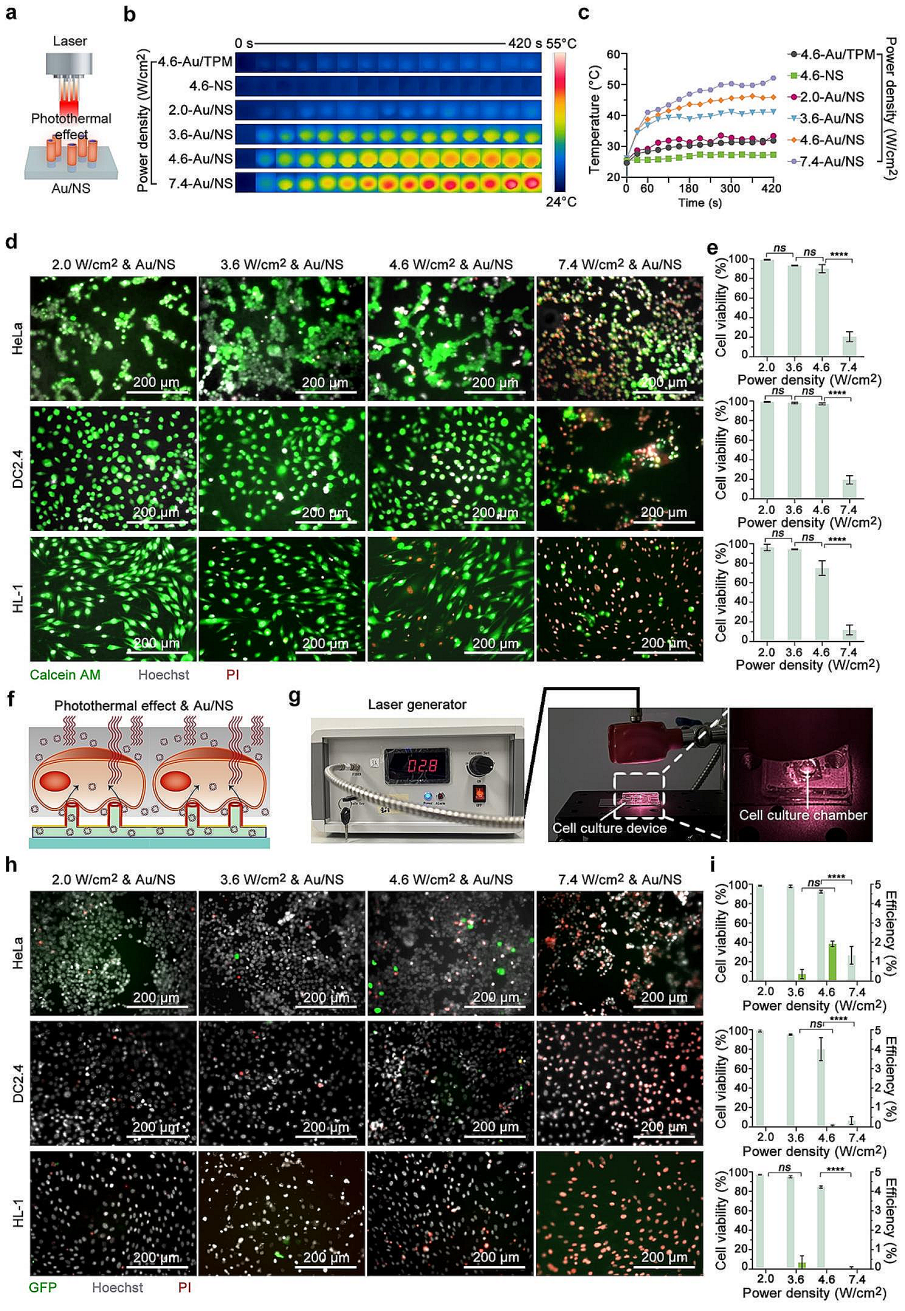
NS coupled with photothermal effect applied to cellular DNA transfection. (**a**) Schematic illustrating the photothermal effect of NS. (**b**) NIR thermal imaging and (**c**) photothermal curves characterizing the photothermal properties of the Au/NS. (**d**) Fluorescent microscopy images capturing the cell condition to various laser intensity applied to the NS. The images display the merged signals from Calcein AM (green), Hoechst (gray), and PI (red). The upper panel represents HeLa cells, the middle panel shows DC2.4 cells, and the lower panel features HL-1 cells. Different laser intensity (2.0, 3.6, 4.6, and 7.4 W/cm^2^) are organized in rows from left to right. (**e**) Cell viability assessment under varied laser intensity conditions based on microscopic images. Mean ± SEM, *n* = 3, One-way ANOVA. (**f**) Schematic of transfection by NS coupled with photothermal effect (Photothermal effect & NS). (**g**) Photograph displaying the cell culture device is exposed to laser irradiation. (**h**) GFP expression observed by microscopy post-transfection via NS coupled with photothermal effect. These images show the merged signals of GFP, Hoechst, and PI, with the rows corresponding to those in panel (**d**). (**i**) Quantification of transfection efficiency and cell viability after pMAX-GFP transfection mediated by NS coupled with photothermal effect, based on the microscopic images. Mean ± SEM, *n* = 3, Two-way ANOVA. Scale bars in all images are 200 μm

Photothermal perforation represents a commonly utilized physical method for intracellular delivery. The following investigation explores the potential application of an additional photothermal effect to aid NS in breaching the cell membrane for DNA transfection. To enhance the photothermal effects of the NS, magnetron sputtering is employed to deposit a gold layer on the surface of the NS (Au/NS). Due to the surface plasmon resonance of gold, Au/NS can absorb energy of infrared light and convert it into heat, thereby elevating their surface temperature (Fig. 5a). The heightened temperature can disrupt the stability of the phospholipid bilayer in the cell membrane, leading to membrane perforation.

To investigate the appropriate laser power density for cellular photothermal perforation, we first assess the photothermal effect of Au/NS through a comparative analysis involving gold-sprayed TPM (Au/TPM) and unsprayed NS. The NS, Au/NS, and Au/TPM are integrated with microfluidic groove into cell culture device. Employing an 808 nm infrared laser with light power densities spanning from 2.0 to 7.4 W/cm², we monitor the temperature dynamic of Au/TPM, NS, and Au/NS throughout a time span of 0 to 420 s. The findings highlight a pronounced photothermal effect in Au/NS, characterized by a fast temperature increase within the initial 60 s of laser exposure, followed by a gradual rise that stabilizes around the 180-s mark. At a low power density of 2.0 W/cm², Au/NS exhibits a temperature increase from the starting point of 25.6 to 31.3 °C after 180 s. At higher power densities of 3.6, 4.6, and 7.4 W/cm², temperatures escalate to 38.8 °C, 43.6 °C, and 46.9 °C, respectively, after the same duration. In contrast, unsprayed NS displays no discernible photothermal effect, and Au/TPM maintains negligible temperature changes, holding at 31.9 °C after 420 s of irradiation at 4.6 W/cm² (Fig. 5b and c, Figure S7a). This stark contrast underscores the pivotal role of the pillar-shaped nanostructure inherent to NS in eliciting the observed photothermal effect.

We further conduct a comprehensive evaluation of the impact of Au/NS-coupled photothermal effects on cell viability across various conditions, with the goal of establishing a secure operational range. Au/NS are integrated with microchannel into cell culture devices and HeLa, DC2.4, and HL-1 cells are culture within the devices. Following 24 h culture, during which cells adhere and spread on the Au/NS, they undergo a 3-min exposure to an 808 nm infrared laser at light power densities of 2.0, 3.6, 4.6, and 7.4 W/cm², respectively. Subsequently, the cells are cultured for an additional 2 h and stained with Calcein AM, Hoechst, and PI for cell viability assessment. Fluorescent microscopy results demonstrate that at a laser power of 2.0 W/cm², cell viability remains unaffected, with the activity of all three cell types maintained above 95%. However, with an increase in laser power to 3.6, 4.6 W/cm², discernible variations in cell activity emerge. HeLa cells exhibit decreased activity to 93.29% and 90.05%, DC2.4 cells maintain activity at 98.02% and 97.06%, while HL-1 cells, being more sensitive to the photothermal effect, experience activity reductions to 94.21% and 75.00%. These findings suggest a gradual impact of the photothermal effect mediated by Au/NS on cell viability with increasing laser power. Upon reaching an applied power of 7.4 W/cm², HeLa cell viability significantly drops to 20.61%, DC2.4 cell viability decreases to 19.61%, and HL-1 cell viability plummets to 11.87% (Fig. 5d and e). This underscores the potential of an excessively strong photothermal effect to induce irreversible cell membrane perforation or trigger cell apoptosis, significantly impacting cell viability.

Subsequent to determining the effects of Au/NS-induced photothermal energy on cell viability, our investigation progresses to assess the effectiveness of Au/NS-coupled photothermal perforation for cell DNA transfection (Fig. 5f). Following a 24-h incubation period of cells on Au/NS, pMAX-GFP is introduced into the cell culture medium and the microchannel 15 min prior to laser irradiation at various power densities for 3 min (Fig. 5g). Cells are then allowed for another 24 h of culture with the presence of pMAX-GFP, followed by staining with Hoechst and PI for further analysis. Characterization results from fluorescent microscopy reveal that coupling Au/NS with 3.6 or 4.6 W/cm² laser irradiation yields GFP expression efficiencies of 0.37% and 1.92% in HeLa cells. Meanwhile, DC2.4 cells display limited transfection, observed only under 4.6 W/cm² condition, with GFP expression efficiencies of 0.05%. HL-1 cells demonstrate a faint transfection efficiency of only 0.34% under 3.6 W/cm² conditions. Notably, all three cell types subjected to Au/NS coupling with a low power density of 2.0 W/cm² or a high-power density of 7.4 W/cm² do not demonstrate successful transfection. The decrease in cell viability after transfection under high power density validate the damage on cell condition (Fig. 5h and i, Figure S7b).

The experimental findings reveal that Au/NS can absorb energy from near-infrared light, converting it into heat. However, this coupling strategy does not significantly enhance intracellular delivery. Similar to the limitations observed in PEI@NS mediated transfection, the photothermal perforation of the cell membrane occurs in the region closely in contact with the substrate, making it difficult for plasmid DNA molecules through this perforated area into the cells. Moreover, the small diffusion coefficient of plasmid DNA in solution, coupled with spatial hindrance, restricts the quantity entering the cytoplasm by free diffusion. Additionally, cells exhibit a narrow tolerance window to the thermal effect, posing a challenge in achieving increased transfection efficiency without compromising cell viability. To enhance the efficiency of intracellular DNA delivery by photothermal perforation, the incorporation of electrophoresis-assisted plasmid DNA migration could be considered to further improve the transfection efficiency.

### Nanostraws coupled with electric field for cellular DNA transfection

**Fig. 6:**
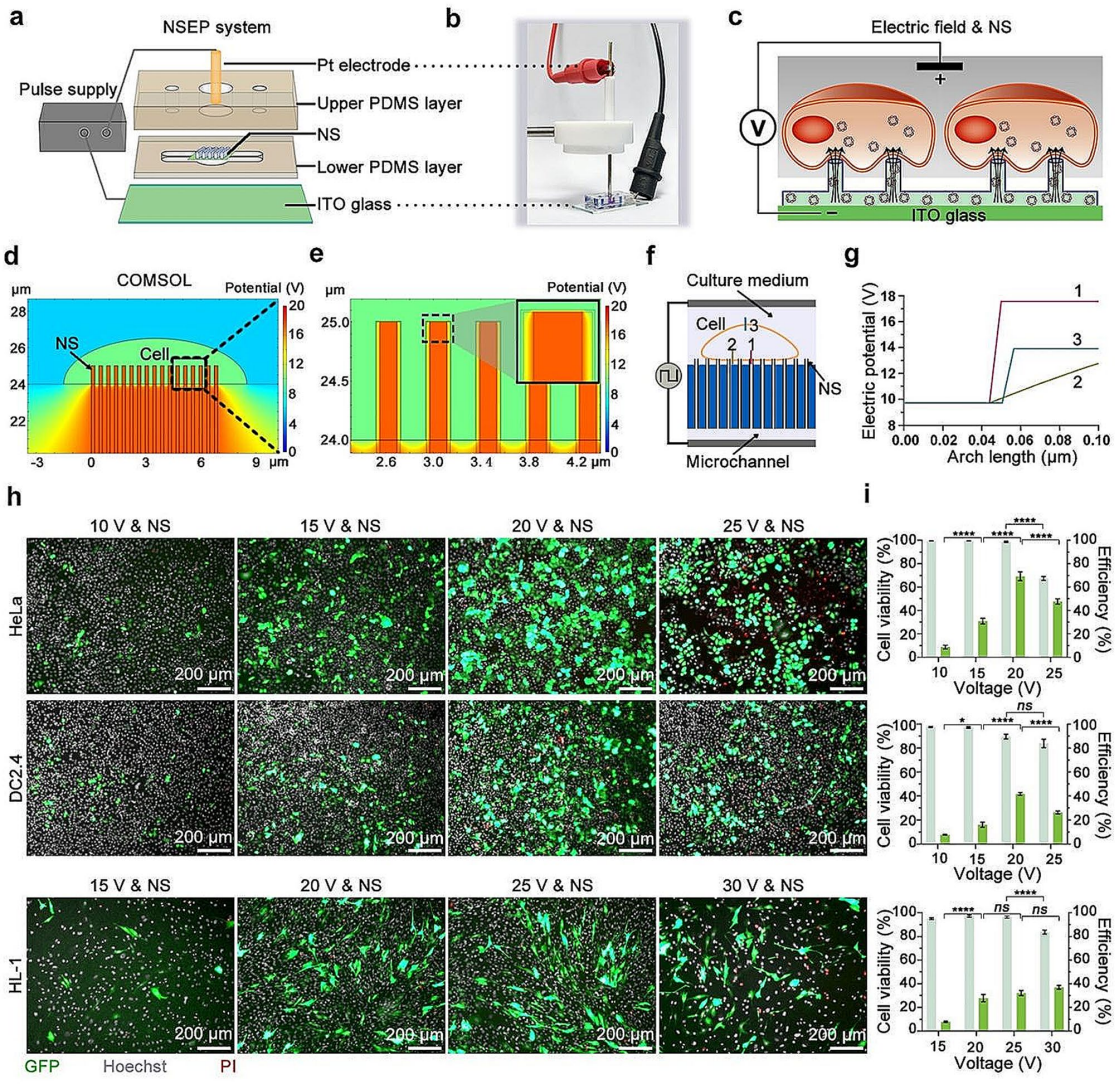
NS coupled with electric field for cellular DNA transfection. (**a**) Components of the NSEP device. The upper PDMS chunk housing the cell culture chamber, the NS, the lower PDMS layer of the microchannel, ITO glass (bottom electrode), a Pt electrode (top electrode) are integrated into the NSEP device. (**b**) Photograph of NSEP device. (**c**) Schematic representation of transfection using NS coupled with electric field (Electric field & NS). (**d**, **e**) COMSOL simulation of electric potential distribution at the cell-NS interface. (**f**) Schematic representation of the 2D model used for COMSOL simulation, featuring three intercept lines marked as 1, 2, and 3 across the cell membrane. (**g**) The electric potential across the cell membrane along the three intercept lines. The horizontal axis, the Arc line, represents the length of the intercept line, while the corresponding Y values from 0 to 0.10 μm represent the electric potential values across the intercept lines from top to bottom as shown in (**f**). (**h**) Fluorescent microscopy images capturing GFP expression 24 h after NS coupled with electric field-mediated transfection. The images merge signals from GFP (green), Hoechst (gray), and PI (red). Upper panel: HeLa cells, middle panel: DC2.4 cells, lower panel: HL-1 cells. Rows from left to right represent different electric voltages (10, 15, 20, 25, and 30 V). Scale bars in all images measure 200 μm. (**i**) Quantitative assessment of transfection efficiency and cell viability following pMAX-GFP transfection via NS coupled with electric field delivery system, as determined from microscopic images. Mean ± SEM, *n* = 5 regions, Two-way ANOVA

After the attempts to couple various physicochemical with NS for cell perforation, we eventually employed an electric field-assisted method to achieve cell membrane electroporation for intracellular delivery of pMAX-GFP. We integrate a layer of indium tin oxide (ITO) glass to replace the ordinary glass located at the bottom as an electrode, along with a platinum (Pt) electrode positioned above the cell culture chamber, constituting a NS-based electroporation (NSEP) device. This configuration forms an electrical circuit encompassing the ITO electrode at the microchannel’s bottom, the solution within the microchannel and inside the NS, the cell culture medium, and the Pt electrode (Fig. 6a and b). The application of electric pulses between these two electrodes facilitates the propagation of the electric field through the conductive solution in microchannel and NS, resulting in a localized effect at the exit tip of the NS. A transmembrane potential of 0.2 ~ 1.5 V represents a critical threshold for cell membrane perforation induced by electric field [40]. In the context of cell membrane tightly adhering to the surface of NS, the localized electric field at the NS-cell membrane interface promotes the disturbance of the stability of the cell membrane structure, ultimately resulting in the perforation. Concurrently, due to the negative charge of DNA molecules, the electric field, with the ITO glass electrode as the cathode and Pt electrode as the anode, prompts DNA molecules to migrate from the microfluidic groove along the NS to the cell membrane through electrophoresis (Fig. 6c).

To gain a deeper insight into the localized effect of the electric field coupled to NS, we construct a 2D COMSOL model based on the geometric structure of the NS array and the cell interaction interface. Specifically, the 2D model consists of a half-ellipse-shaped hollow cell membrane shell, measuring 6 nm in thickness (Figure S8 and Table S1). This membrane shell embraces the NS. The NS are filled with an electrically conductive cell culture medium, while the microchannel at the bottom is also filled with a cell culture medium (Figure S8 ). Using the AC/DC Module in a steady-state regimen in COMSOL for simulation calculations [16], we apply an initial voltage (V_0_) of 20 V. The simulation modeling of the electric potential distribution reveals a concentration of high potential on the NS, with a noticeable decline after traversing the cell membrane above them (Fig. 6d and e). Similarly, the norm intensity of the electric field is primarily accumulated at the interface between the NS and the cell membrane, intensifying to maximum strength at the top of the NS (Figure S9a,S9b). The localized electric field generated by the NS, combined with a significant difference in potential between the external and internal aspects of the cell, facilitates the targeted accumulation of energy needed to overcome the perforation energy barrier in that specific region. Meanwhile, the majority of other regions experience only weak electric fields, ensuring the cell’s safety. We further scrupulously analyze the electric field norm distribution and the transmembrane potential at different regions, encompassing the interface between the cell membrane and NS, as well as areas within the cell not contact to NS either proximal or situated at a distance from the NS. The locations of the three regions are illuminated with three lines in the schematic diagram (Fig. 6f). With the V_0_ setting at 20 V, the transmembrane voltage of the cell membrane in contact with the NS reaches 7.9 (Line 1), whereas the transmembrane voltage of the cell region at the proximal end (Line 2) or farther away from the NS (Line 3) remains below 5 V (Fig. 6g). Additionally, the position indicated in line 1 demonstrates the most substantial variation in electrical field strength when compared to the other two sections (Figure S9c). This outcome signifies that NS effectively concentrate the electric field at the tip exit, enabling the cell membrane in contact to attain the critical transmembrane voltage condition for perforation. Conversely, the cell membrane not in contact with the NS withstands minimal transmembrane voltage, averting extensive permeabilization and preserving the integrity of most cell membranes, thus ensuring cell health.

Based on our previous research, we have gained insights into the optimal range of voltage conditions for cell transfection [41]. In this study, we employ an electric field-coupled NS system with voltage ranging from 10 to 30 V for cell membrane perforation. Leveraging the electrophoretic mobility of plasmid DNA in the presence of an electric field, where ITO glass functions as the cathode and a Pt electrode as the anode, plasmids undergo directed migration from the lower microchannel to the immediate vicinity of cells. Furthermore, we systematically evaluate the effectiveness across a spectrum of voltage conditions for cellular DNA transfection. Following a 24-h incubation of cells within the NSEP device, a solution containing 0.1 µg/µl pMAX-GFP is introduced into the lower microchannel prior electroporation. Subsequently, electric pulses spanning a range from 10 to 30 V, with a pulse frequency of 20 Hz, pulse width of 200 µs, and a total pulse duration of 30 s, are applied. Following that, cells are cultured for an additional 24 h, followed by staining with PI and Hoechst for microscopy observation to determine transfection efficiency and cell viability.

The results demonstrate that coupling NS with voltage conditions of 10, 15, 20, and 25 V yields GFP expression efficiencies of 8.74%, 30.94%, 69.33%, and 47.72% for HeLa cells, with corresponding cell viability of 99.61%, 99.69%, 98.73%, and 67.37%, respectively. DC2.4 cells demonstrate GFP expression efficiencies of 7.74% (10 V), 16.11% (15 V), 41.76% (20 V), and 26.25% (25 V), respectively. Their cell viability remains above 95% under voltages below 15 V but slightly decreases to 89.57% and 83.86% when applying 20 and 25 V to the NS integrated system. Similarly, HL-1 cells subjected to NS coupling under voltage conditions of 15, 20, 25, and 30 V, exhibit GFP expression efficiencies of 7.87%, 27.94%, 32.25%, and 37.05%. HL-1 cell viability does not significantly decrease after NS combined electroporation under voltage conditions of 15, 20, and 25 V, while a palpable reduction to 83.59% occurs under the higher voltage condition of 30 V (Fig. 6h and i, Figure S10). These findings underscore the notion that subjecting cells to NS with an external electric field for perforation under optimized conditions significantly amplifies the efficiency of pMAX-GFP transfection with minimal cell damage. Excessively high voltage applied for NS coupling can cause irreversible damage to cells, negatively impacting their viability. Across HeLa, DC2.4, and HL-1 cell lines, efficient and high-quality DNA transfection is achievable under low-voltage conditions, with the most favorable transfection effects observed at 20 V, 20 V, and 25 V, respectively. It is noteworthy that beyond these optimal voltage settings, further increases in voltage fail to enhance cell transfection efficiency, while concurrently leading to a noticeable decline in cell viability.

The robust transfection efficiency mediated by the NS-electric field coupling strategy may be due to several factors. Firstly, the electric field is confined to the nanochannels of the NS, creating a nanoscale electric field localized specifically on the cell membrane surface above the NS. This results in a significant electric potential difference between the interior and exterior of the cell membrane, even under low-voltage electrical pulse conditions. Secondly, the specific electrode configuration, with a Pt electrode as the anode and an ITO glass electrode as the cathode, accommodates the negatively charged DNA molecules, facilitating their efficient migration along the NS towards the cells through electrophoresis. Thirdly, prior investigations have demonstrated that cell membrane perforations induced by this method typically undergo complete repair within a maximum of 5 min, thereby showcasing minimal cytotoxic effects. Consequently, this ensures the successful expression of DNA plasmids subsequent to their entry into the cells.

**Fig. 7:**
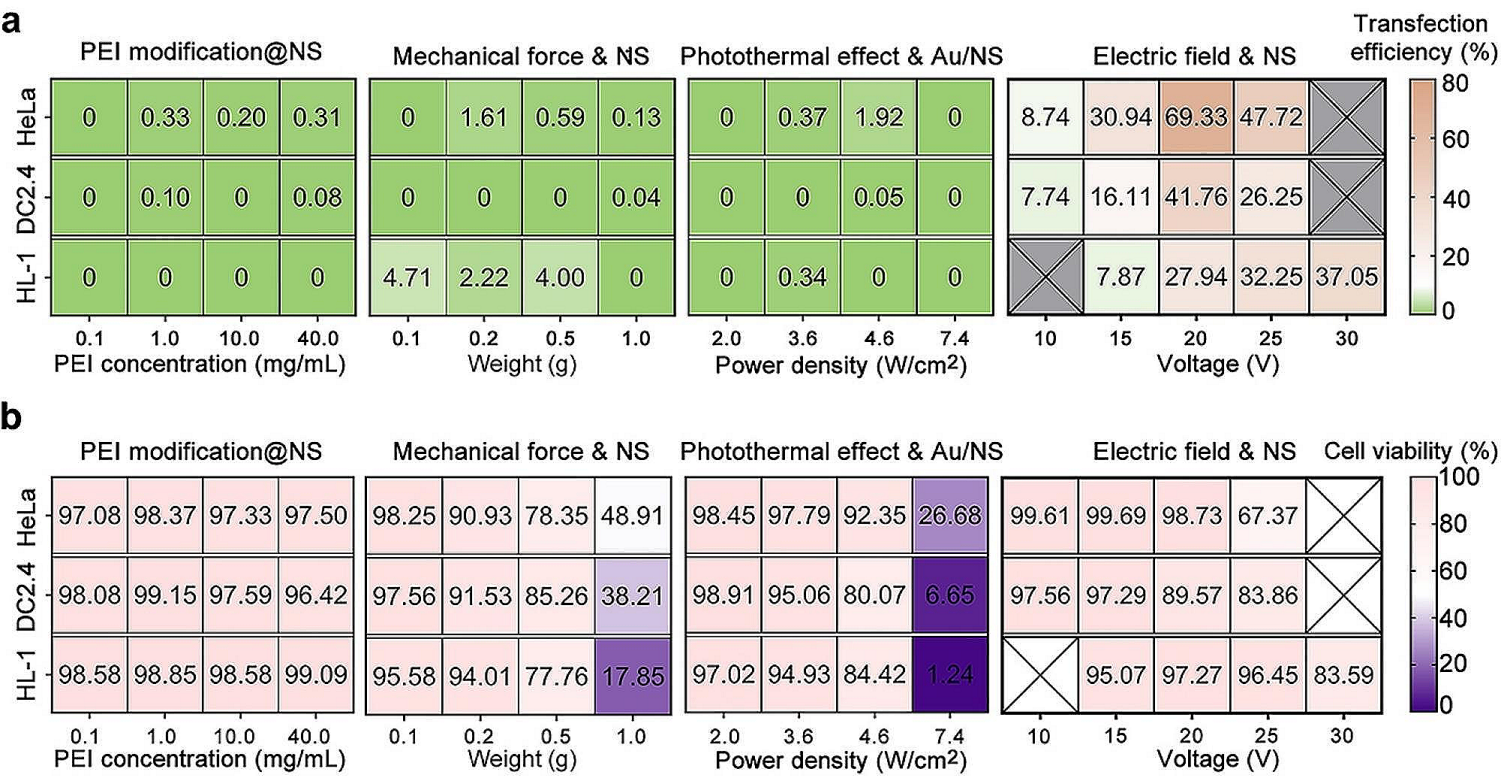
Heatmap depicting transfection efficiency and cell viability 24 h after intracellular pMAX-GFP delivery through NS coupled with four distinct physicochemical perforation methods. (**a**) Comparative analysis of transfection efficiency mediated by four coupling strategies. (**b**) Cell viability analysis after transfection using four coupling strategies. Crossed squares indicate no testing data

In our aforementioned study, we systematically compare the optimal efficiency of cellular DNA transfection achieved by NS coupling with PEI modification, mechanical forces, photothermal effects, and electric filed. Additionally, we conduct an analysis of cell viability under these conditions. The results reveal that, for HeLa cells, the optimal transfection efficiency with NS-coupled positively charged PEI modification is limited to 0.33%, though cell viability remains relatively high at 98.37%. NS coupling with mechanical forces achieves a modest transfection efficiency of 1.61%, with cell viability at 90.93%. Similarly, the optimal transfection efficiency mediated by NS coupling and photothermal effects is 1.92%, with cell viability at 92.35%. Notably, NS coupling with electroporation demonstrates an impressive optimal transfection efficiency of 69.33%, while maintaining high cell viability at 98.73%. Clearly, the transfection efficacy of NS coupling with electroporation surpasses that of the other three physicochemical techniques. For DC2.4 cells, the optimal transfection efficiency mediated by NS-coupled PEI modification is 0.1%, with cell viability at 99.15%. Unfortunately, NS coupling with mechanical forces fails to achieve transfection, even under high-pressure conditions, significantly impacting cell viability. In the context of NS coupling with photothermal effects, the optimal transfection efficiency is a mere 0.05%, with cell viability at 80.07% under the laser power density of 4.6 W/cm^2^. In contrast, NS coupling with electroporation in DC2.4 cells demonstrates a remarkable optimal transfection efficiency of 41.76%, coupled with a cell viability of 89.57%. This underscores the exceptional performance of NS coupling with electroporation in terms of transfection efficacy, compared to the other three physicochemical techniques. Similarly, for HL-1 cells, NS-coupled PEI modification fails to achieve successful transfection, although with no significant impact on cell viability. NS coupling with mechanical forces achieves an optimal transfection efficiency of 4.71%, maintaining a high cell viability of 95.58%. NS coupling with photothermal effects achieves an optimal transfection efficiency of 0.34%, with cell viability at 94.93%. Significantly, NS coupling with electroporation once again demonstrates superior transfection efficacy, with an optimal transfection efficiency of 32.25%, coupled with a cell viability of 96.45% (Fig. 7a and b). Overall, these research findings underscore the universality and superiority of NS coupling with electroporation across different cell lines. Two key factors contributing to their outstanding performance are rooted in the localized electric field generated by NS, resulting in membrane perforation, and the electrophoresis of DNA molecules from the lower microchannel to the cellular environment under the customized electric field direction. Meanwhile, NS-coupled electric field-mediated transfection offers several advantages over traditional transfection methods. The NS-electric field facilitates direct entry of plasmids into the cytoplasm. This bypasses potential degradation that may occur through endocytosis pathways, which is a limitation of chemical carriers. While bulk electroporation, a widely used method, can also deliver plasmids directly into the cytoplasm, it is prone to drawbacks such as cell damage from high-voltage electrical pulses and uneven transfection efficiency across cell populations. In contrast, the low voltage utilized in NS-coupled electric field strategies minimizes cell toxicity and promotes rapid cell repair. Additionally, the design of microfluidic devices employed in NS-coupled electric field approaches enables precise and repetitive delivery, further enhancing the efficacy and versatility of this transfection method. Therefore, electric field coupling forms constitute the prioritized modality for further enhancing the efficiency of NS-mediated DNA transfection.

## Conclusion

In summary, our investigation revolves around integrating an array of NS with four physicochemical perforation techniques, including PEI modification, mechanical forces, photothermal effects, and electric fields, aiming to enhance the efficiency of cell membrane perforation and consequently promote DNA transfection. Detailed investigations are conducted on representative cell lines, namely HeLa, DC2.4, and HL-1, with a specific emphasis on optimizing transfection efficiency through adjustments in applied intensity for each physicochemical coupling forms. The results validate that NS face challenges in penetrating the cell membrane solely relying on the inherent forces of the cells. Despite the application of external power, involving PEI modification, mechanical forces, and photothermal effects, NS demonstrate relatively modest improvements in DNA transfection. Specifically, coupling with mechanical forces yields non-uniform transfection outcomes, and coupling with photothermal effects displays a limited effective window, potentially leading to cell death. Moreover, the influence of PEI modification on promoting NS-mediated cell membrane perforation is notably weak. In stark contrast, the amalgamation of electric fields with NS markedly enhances their efficiency in DNA transfection, consistently showcasing improved effects across the three cell lines. In conclusion, our research underscores NS as a versatile material platform adept at flexible coupling with diverse physicochemical perforation methods. The exploration in this study paves the way for optimizing the forms and conditions of additional physicochemical perforation techniques, thereby improving the efficiency of NS-mediated DNA transfection into cells. This work not only showcases the feasibility of highly efficient cell transfection using micro-nano devices but also holds promising implications for the development of safe and efficient transfection technologies applicable across various cell types. The ongoing advancement of the transfection platform based on NS is poised to facilitate its widespread utilization in cellular and gene therapy technologies.

## Materials and methods

### Cell culture

HeLa (ATCC) and DC2.4 (ATCC) cells are cultured in DMEM (Corning) supplemented with 10% heat-inactivated fetal bovine serum (FBS, Excell), 100 U/mL penicillin, 100 µg/mL streptomycin (Gibco), and 1% non-essential amino acid (NEAA, HyClone). HL-1 (ATCC) cells are cultured in MEM (Gibco) supplemented with 10% heat-inactivated fetal bovine serum (FBS, Excell), 100 U/mL penicillin, 100 µg/mL streptomycin (Gibco), and 1% NEAA (HyClone). All cells are maintained in a tissue culture incubator at 37 ℃ plus 5% CO_2_ with saturating humidity. Upon the confluence reaching 80%, HeLa, DC2.4, and HL-1 cells undergo passaging using trypsin (Corning). Specifically, the cell culture medium is discharged, followed by washing with PBS. Subsequently, a small amount of pre-warmed trypsin is added, and the cells are incubated for several minutes in the tissue culture incubator. The trypsinization process is neutralized with an equal volume of complete cell culture medium, followed by centrifugation and resuspension of cells in fresh culture medium.

### The fabrication of NS

A 25-µm thick TPM with pore size of 200 nm and density of 2 × 10^7^ nanochannels per cm^2^ (it4ip) serves as the foundational template. The porous TPM is fabricated by bombardment with heavy ion beams. The particles create tracks as they pass through the membrane, forming cylindrical channels with precise size, shape, and distribution corresponding to the original tracks. The membrane initially undergoes ALD to deposit a ~ 20-nm thick Al_2_O_3_ layer on both their surface and within the nanochannels’ walls. The ALD procedure involves the sequential introduction of triethylaluminum (Al(C_2_H_5_)_3_) and water vapor into the reaction chamber. This chemical reaction is represented by the equation: 2Al(C_2_H_5_)_3_ + 3H_2_O → Al_2_O_3_ + 6C_2_H_6_. Following 120 cycles, a uniform Al_2_O_3_ layer forms on both the TPM surface and within the nanochannel walls. Subsequently, the chamber is purged with nitrogen to eliminate any residual unreacted Al(C_2_H_5_)_3_ and the generated C_2_H_6_. Following this, the Al_2_O_3_ layer on the surface of the TPM is selectively removed through RIE, exposing the underlying TPM surface. Further, O_2_ plasma etching is employed to remove a certain height of the exposed TPM, revealing the Al_2_O_3_ nanostructure within the nanochannels, forming an array of NS The power for the O_2_ plasma etching is set at 100 W, and precise control of the etching time results in the attainment of NS with a consistent height of 1 μm.

### SEM characterization

DCs are cultured on the FN-coated NS for 24 h. The cells are then fixed in a 2.5% glutaraldehyde solution, followed by a series of sequential dehydration steps, involving immersion in ethanol aqueous solutions with increasing concentrations (30, 50, 70, 90, 100%). The next dehydration step involves substituting acetone for the 100% ethanol, and the samples are then transferred to isoamyl acetate. Following this, a critical-point drying (Leica EM CPD300) process is performed to ensure complete dehydration. The samples are sputtered with a thin layer of Au (KYKY GVC-2000) for SEM imaging. The TPM and the NS are directly sputtered with a thin layer of Au before SEM characterization. The SEM images are captured using a field emission scanning electron microscope (Zeiss SUPRA 60).

### Cell culture devices construction

Initially, the NS integrate with a microfluidic design to construct NS-based perforation devices for cell culture. In summary, the device comprise a cell-culture chamber and a microfluidic channel made of PDMS, an intermediate layer of NS, and a glass substrate. The fabrication process commences by thoroughly blending PDMS monomer and curing agent (Dow Corning) at a weight ratio of 10 (monomer) to 1 (curing agent). The mixture is then poured into a mold and cured at 80 °C for a minimum of 30 min. The PDMS chunk of the cell culture chamber measures 30 mm × 20 mm × 5 mm, with a central chamber of 5 mm in diameter specifically designed for cell culture. Additionally, two through-holes, each with a diameter of 1.5 mm, are positioned on either side of the cell culture chamber, connected to the microchannel, serving as injection ports for the delivery of solutions. The microfluidic layer, with dimensions of 30 mm × 20 mm × 0.5 mm, encompasses an elongated channel measuring 15 mm in length and 1 mm in width. The two terminals of the microchannel also feature two circular holes with a diameter of 1.5 mm, aligning with that beside in the cell culture chamber. The solution introduced through the holes adjacent to the cell culture chamber can flow into the bottom microchannel. To assemble all the components, the PDMS layer containing the microfluidic channel is first stuck onto glass substrates with the uncured PDMS and then heated at 80 °C for 15 min. The TPM housing NS is attached onto the PDMS layer, covering the microfluidic channel. Finally, the PDMS section of the cell culture chamber is carefully attached to the lower PDMS layer, with the NS in the middle of the two PDMS layers. The established cell culture devices are sterilized with UV light for 1 h and the NS are immersed in a solution containing 100 ng/mL Fn (Sigma) and incubated at 37 °C for 6 h, followed by washing with PBS before cell culturing. Subsequently, a certain number of cells are seeded in the upper chamber and given a 24-h period for adhesion or growth. For HeLa and DC2.4, the seeded cell numbers are 8000, while for HL-1 the cell number is 5000. Unless stated otherwise, the cell culture process in the device consistently adheres to this protocol.

### Cell viability assessment

HeLa, DC2.4, and HL-1 cells cultured within cell culture devices for either 6 or 24 h undergo staining procedures to assess their growth characteristics and cell viability. A co-staining approach with Calcein-AM (Invitrogen), PI (Sigma), and Hoechst 33,342 (Invitrogen) enables the quantification of live cells, dead cells, and total cells, respectively. A working solution is prepared by diluting the dyes in cell culture medium, resulting in concentrations of 6 µM for Calcein-AM, 5 µM for Hoechst, and 0.5 µM for PI. The working solution is introduced to ensure comprehensive coverage of the cells. Following a 15-min incubation period at 37℃, the working solution is exchanged with fresh culture medium before microscopy observation.

### Intracellular DNA delivery by NS alone

HeLa, DC2.4, and HL-1 cells are seeded onto Fn-coated NS within cell culture devices for 24 h, then a solution containing pMAX-GFP is introduced into the cell culture medium and the lower microchannel at a concentration of 0.1 µg/µL. Subsequently, the cells are cultured in the incubator for additional 24 h. Afterward, the cells are stained with PI and Hoechst 33,342, following a protocol similar to the one described previously, before microscopy observation. Plasmid pMAX-GFP is procured at a concentration of 1 µg/µL from commercially available cell transfection kits (Lonza, Cat No.: V4XP-3032).

### NS coupled with PEI modification

The TPM housing an array of NS is affixed to the substrate of cell culture plates. A solution of 10 mM silane coupling agent KH-560 (Sigma) is added to cover the TPM and incubate at room temperature (RT) for 24 h. Polyethyleneimine (PEI, Mw ~ 800 g/mol) is diluted in double-distilled water (ddH_2_O) to achieve final concentrations of 0.1, 1.0, 10.0, and 40.0 mg/mL, respectively. The diluted PEI solutions are then introduced to cover the TPM, followed by overnight incubation at RT. For the formation of PEI-Cy5@NS, Cy5 first undergoes an activated ester reaction with N-hydroxysuccinimidyl ester, thereby forming Cy5 with an activated carboxyl group (Cy5-NHS). Cy5-NHS, which reacts with amidogen on PEI, is then added to PEI@NS at a concentration of 20 µM and incubated for 24 h at RT before fluorescence imaging. A thorough washing step with PBS and ddH2O is carried out after each reaction.

HeLa, DC2.4, and HL-1 cells are seeded on Fn-coated PEI@NS and allowed to incubate for 24 h. Subsequently, the cell viability is assessed using live/dead staining. For cellular DNA transfection experiments, following a 24-h culture period on the PEI@NS, pMAX-GFP is introduced into the cell culture medium at a concentration of 0.1 µg/µL. The cells are then cultured at 37 °C for an additional 24 h to allow for successful GFP expression before microscopy imaging.

### NS coupled with mechanical force

HeLa, DC2.4, and HL-1 cells are initially seeded on conventional 48-well cell culture plate (Greiner Bio-One) for 24 h to promote adhesion. Subsequently, the TPM housing NS is attached to a coverslip with the radius of 4 mm (Solarbio), and then meticulously positioned in an inverted orientation onto the adherent cell surface. This arrangement ensures the vertical contact of NS with the cells. Virous degrees of external mechanical force is applied using cured PDMS onto the coverslip. Specifically, un-crosslinked PDMS is prepared with precise weight measurement before curing, ensuring the formation of solid PDMS with specific weight properties. The total weight includes the cured PDMS plus the coverslip weights, with values of 0.1, 0.2, 0.5, and 1.0 g, respectively. Following a 12-h exposure to these mechanical forces, the PDMS weights and the NS adhered to the coverslip are carefully removed. Subsequently, the cells are subjected to live/dead staining for viability assessment.

In cellular DNA transfection experiments, after an initial 24-h seeding period on conventional cell culture plates, a controlled external force is applied to the cells. As detailed in the context, this force is sustained for a duration of 12 h before being carefully removed. Afterward, pMAX-GFP is added to the cell culture medium at a concentration of 0.1 µg/µL, and the cells are cultured for another 24 h for GFP expression. Subsequently, cell viability and transfection efficiency are evaluated by microscopy imaging after staining.

### NS coupled with photothermal effect

Firstly, a gold layer is deposited onto the surfaces of both the NS and the TPM utilizing of an automated magnetron ion sputtering instrument (KYKY GVC-2000). The thickness of the gold layer is determined by controlling the sputtering duration. To maintain transparency for microscopy imaging of the TPM with/without NS, a minimal yet efficient thickness of the gold layer exhibiting a significant photothermal effect is deemed optimal in this context. The sputtering current is consistently set at 30 mA, and extending the sputtering duration results in a thicker gold layer. Upon comparing the photothermal effects of gold layers sputtered for 3 and 5 min, the latter exhibits superior performance while maintaining the transparency of TPM. Then the NS, Au/NS or Au/TPM is integrated into cell culture devices equipped with microfluidic channel.

To validate the photothermal effects, 60 µL PBS is introduced into the cell culture chamber, and the cell culture device is positioned 3 cm beneath a laser probe to enable irradiation of the entire chamber. A laser generator (Laserwave LWIR808-2 W) emitting continued light at 808 nm is employed to provide laser power, and a power meter (Laserwave LW-P10W-A) is utilized for precise laser power detection. The power density is determined by dividing the light power by the area of the light spot. The NS, Au/NS or Au/NPM within the cell culture chamber is subjected to the laser irradiation at varying power densities (2.0, 3.6, 4.6, and 7.4 W/cm²). Temperature dynamics of NS, Au/NPM, and Au/NS are monitored over a duration of 0 to 420 s using an infrared thermal imaging camera (FOTRIC).

HeLa, DC2.4, and HL-1 cells are seeded on Au/NS within cell culture chamber, and after 24 h, these cells undergo laser irradiation for 3 min at various power densities (2.0, 3.6, 4.6, and 7.4 W/cm²). Subsequently, the cells are cultured continuously for an additional 2 h before undergoing live/dead assessments.

In the context of cellular DNA transfection experiments, pMAX-GFP is introduced into the cell culture medium, simultaneously loaded into the lower microfluidic channel at a concentration of 0.1 µg/µL for 15 min preceding laser irradiation. Subsequently, cells undergo laser irradiation for 3 min at various power densities (2.0, 3.6, 4.6, and 7.4 W/cm²). After an additional 24 h of culture, cellular samples are stained and then subjected to microscopy for transfection efficiency and cell viability assessments.

### NS coupled with electric field

The cell culture device designed for the NS coupled with an electric field scenario comprises an upper PDMS layer with cell-culture chamber, a central TPM with NS, a lower PDMS layer housing a microfluidic channel, and an ITO glass electrode, as illustrated in Fig. 6a. All components are assembled to NSEP devices as previous description. The ITO glass functions as the bottom electrode in the NSEP devices. For electroporation, a waveform generator (Rigol) and a voltage amplifier (Agitek) are employed to generate electric pulses. A Pt electrode, serving as the top electrode, is immersed into the cell culture medium to establish an electrical circuit with the ITO glass. To facilitate cellular DNA delivery, the Pt electrode functions as the anode, while the ITO glass serves as the cathode.

In the context of cellular DNA transfection experiments, a solution of pMAX-GFP is loaded into the lower microchannel at a concentration of 0.1 µg/µL, 3 min prior to electroporation. A square-wave electric pulse ranging from 10 to 30 V with a duration time of 200 µs, frequency of 20 Hz, and a total period of 30 s is applied between the ITO glass and Pt electrodes. The pMAX-GFP solution is removed 15 min post-electroporation, and the cells are subsequently cultured at 37 ℃ for another 24 h with fresh culture medium before cell staining and microscopy imaging.

### COMSOL simulation

The AC/DC Module of the COMSOL Multiphysics finite-element-analysis software (COMSOL Inc) is used to stimulate the electric field and electric potential distribution upon the application of an electric pulse between the ITO glass and the Pt electrode. The 2D model is designed to mimic a cell situated on NS, with a conductive cell culture medium in the upper layer and a conductive solution in both the lower microfluidic channel and within the NS. To simplify the model, the cell membrane is represented as a half-ellipse with a thickness of 6 nm, mirroring its actual dimensions, and is assigned a conductivity of 0.000001 S/m. The TPM with insulating properties, is assigned a conductivity value of 0.000001 S/m. The NS, filled with a solution containing nucleic acid molecules, demonstrate enhanced electrical conductivity attributed to the presence of freely dispersed ions. Similarly, the upper conductive cell culture medium and the solution within the NS and lower microfluidic channels are assigned a conductivity of 0.2 S/m. To exclude the influence of intracellular organelles, the electrical conductivity values of the internal cell environment are standardized to 0.2 S/m due to the conductive cytoplasm, with a particular focus on the properties of the cell membrane within this model. Specific parameters are established in the model to faithfully depict the electric field scenario associated with NS coupling. The upper cell culture medium is defined with a height of 3 mm, and the lower microfluidic channel solution is set to a height of 1 mm. In the model, the starting point for the potential is designated at the bottom of the lower microfluidic channel solution, initialized at 20 V. Conversely, the top of the upper cell culture medium is defined as the reference point, with an assigned potential value of zero.

The COMSOL simulation model employed in this study incorporates the following control equations:

$$\nabla \cdot{\rm{J }} = {\rm{ }}0$$

J = бE

$${\rm{E }} = {\rm{ }} - \nabla {\rm{V}}$$

Where J is the transmitted current density, б is the electrical conductivity, E is the electric field strength, and V is the electric potential. By combining these three equations, Laplace’s equation for the global calculation of electric field and potential is derived:

∇(б·V) = 0

#### Fluorescent microscopy imaging and analysis

Microscopic imaging is conducted using the fluorescence microscope (Leica DMi8 or Olympus CKX53 or Olympus FV3000). ImageJ software is utilized to process microscopy images. Specifically, for cell viability analysis, the images of cells labeled with Hoechst dye are converted to 8-bit format, and then the threshold is fine-tuned and particle analysis is performed to count all the cells. The number of Hoechst-labeled cells is denoted as N_Hoe_. The cells labelled with PI undergo the same analysis procedure, and recorded as N_PI_. Alternatively, manual counting is performed if the cell borders are unclear. The cell viability is calculated using the following equation: Cell viability = N_PI_ ÷ N_Hoe_ × 100%.

In the context of transfection efficiency analysis, the quantification of GFP-positive cells and total cells is conducted by ImageJ software as the detailed method described above, denoted as N_GFP_ and N_Hoe_. The delivery efficiency of pMAX-GFP is calculated using the following equation:

Delivery efficiency = N_GFP_ ÷ N_Hoe_ × 100%.

### Electronic supplementary material

Below is the link to the electronic supplementary material.

Supplementary Material 1

## Data Availability

All data generated and analyzed during this research are included in this published article and its supporting information file.

## Declarations

### Supporting information

Diameter and height measurements of nanochannels and NS; the components and dimensions of the cell culture device; experimental data of the fluorescence microscopy imaging; 2D simulation model with a table of the parameters; the graph of the electric field in the simulation model.
