# Supplementary material for: Coupling of nanostraws with diverse physicochemical perforation strategies for intracellular DNA delivery
Source: J Nanobiotechnology. 2024 Mar 26;22:131. doi: 10.1186/s12951-024-02392-w (PMC10964692; doi:10.1186/s12951-024-02392-w)
Supplement: Supplementary file 1 — Supplementary Material 1 [file 12951_2024_2392_MOESM1_ESM.docx]

**Supporting Information**

**Coupling of Nanostraws with Diverse Physicochemical Perforation Strategies for Intracellular DNA Delivery**

Juan Jiang^1, +^, Jing Liu^1, 2 +^, Xinmin Liu^1, +^, Xingyuan Xu^2^, Zhengjie Liu^2^, Shuang Huang^2^, Xinshuo Huang^2^, Chuanjie Yao^2^, Xiafeng Wang^1^, Yixin Chen^3^, Hui-jiuan Chen^2^*, Ji Wang^1^*, Xi Xie^1, 2^*

1. Institute of Precision Medicine, The First Affiliated Hospital, Sun Yat-sen University, 510080 Guangzhou, People’s Republic of China

2. State Key Laboratory of Optoelectronic Materials and Technologies, Guangdong Province Key Laboratory of Display Material and Technology, School of Electronics and Information Technology, Sun Yat-Sen University, 510006 Guangzhou, People’s Republic of China.

3. Sun Yat-sen University Zhongshan School of Medicine, 510080 Guangzhou, People’s Republic of China.

+ J.J., J.L., and X.L. contributed equally to this work.

*e-mail: [chenhuix5@mail.sysu.edu.cn](mailto:chenhuix5@mail.sysu.edu.cn); [wangj683@mail.sysu.edu.cn](mailto:wangj683@mail.sysu.edu.cn); [xiexi27@mail.sysu.edu.cn](mailto:xiexi27@mail.sysu.edu.cn)


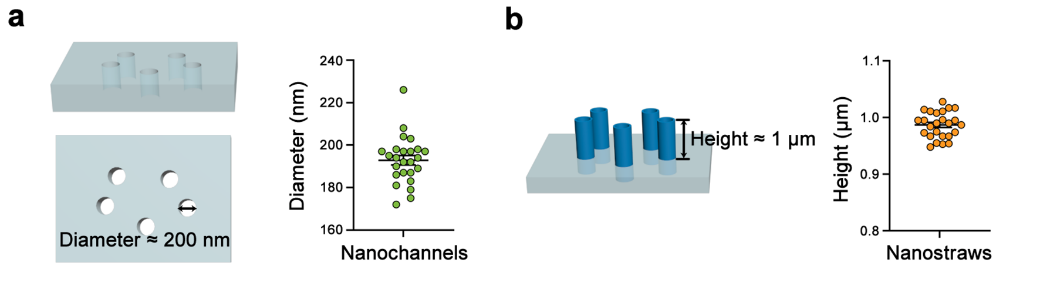


**Figure S1.** Physical parameters of nanochannels and NS. (a) Schematic representation (left) and statistical quantification (right) illustrating the diameters of nanochannels embedded in the TPM. Mean ± SEM, n = 25. (b) Schematic depiction (left) and statistical quantification (right) presenting the heights of NS. The measurements of nanochannel diameter and the height of NS are conducted based on SEM images. Mean ± SEM, n = 25.


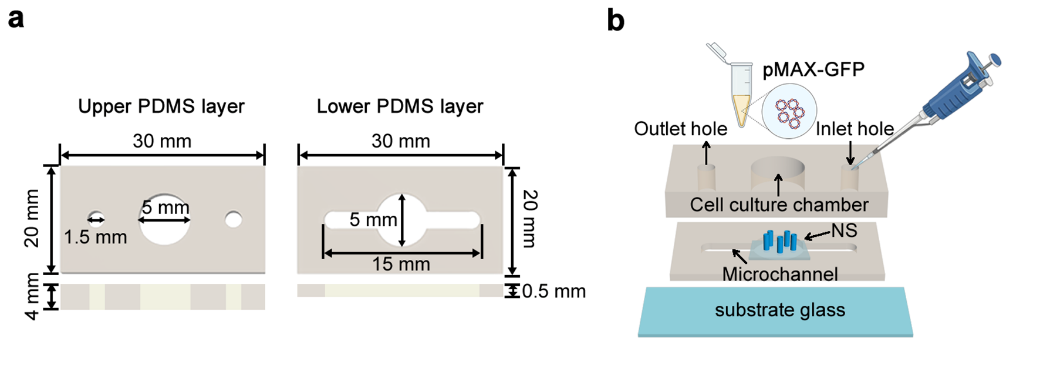


**Figure S2.** Construction of the cell culture device for NS-based delivery. (a) The dimensions of the upper PDMS layer accommodating the cell culture chamber, and the lower PDMS layer housing the microchannel. (b) Illustration outlining the construction and assembly of the cell culture device.


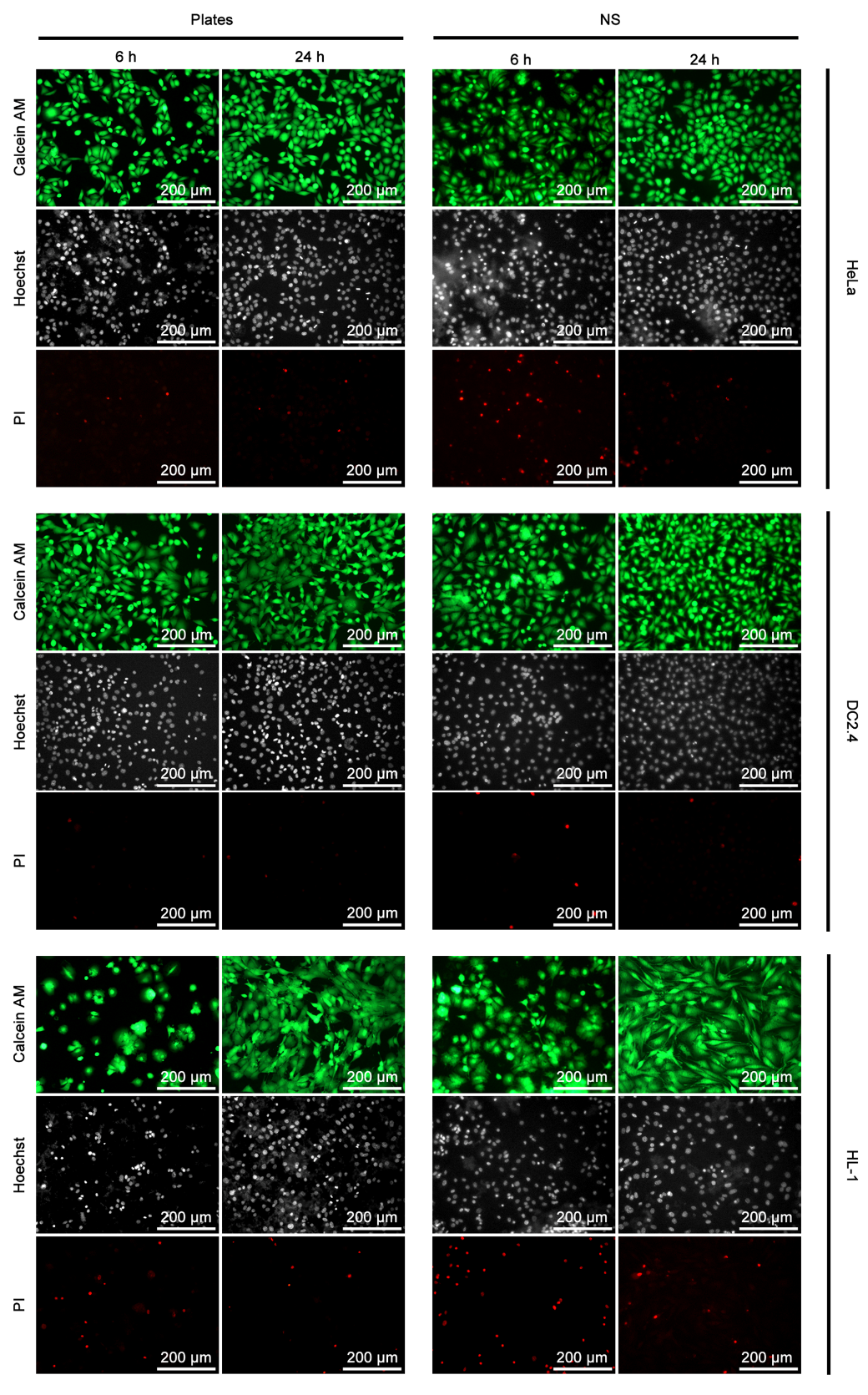


**Figure S3.** Cell viability assessment after culturing on the NS. Representative single-channel fluorescent microscopy images corresponding to Figure 2a are presented. Calcein AM (green) labels the cytoplasm of live cells, Hoechst (gray) stains the nucleus, and PI (red) is employed to distinguish dead cells. Scale bars in all images are 200 µm.


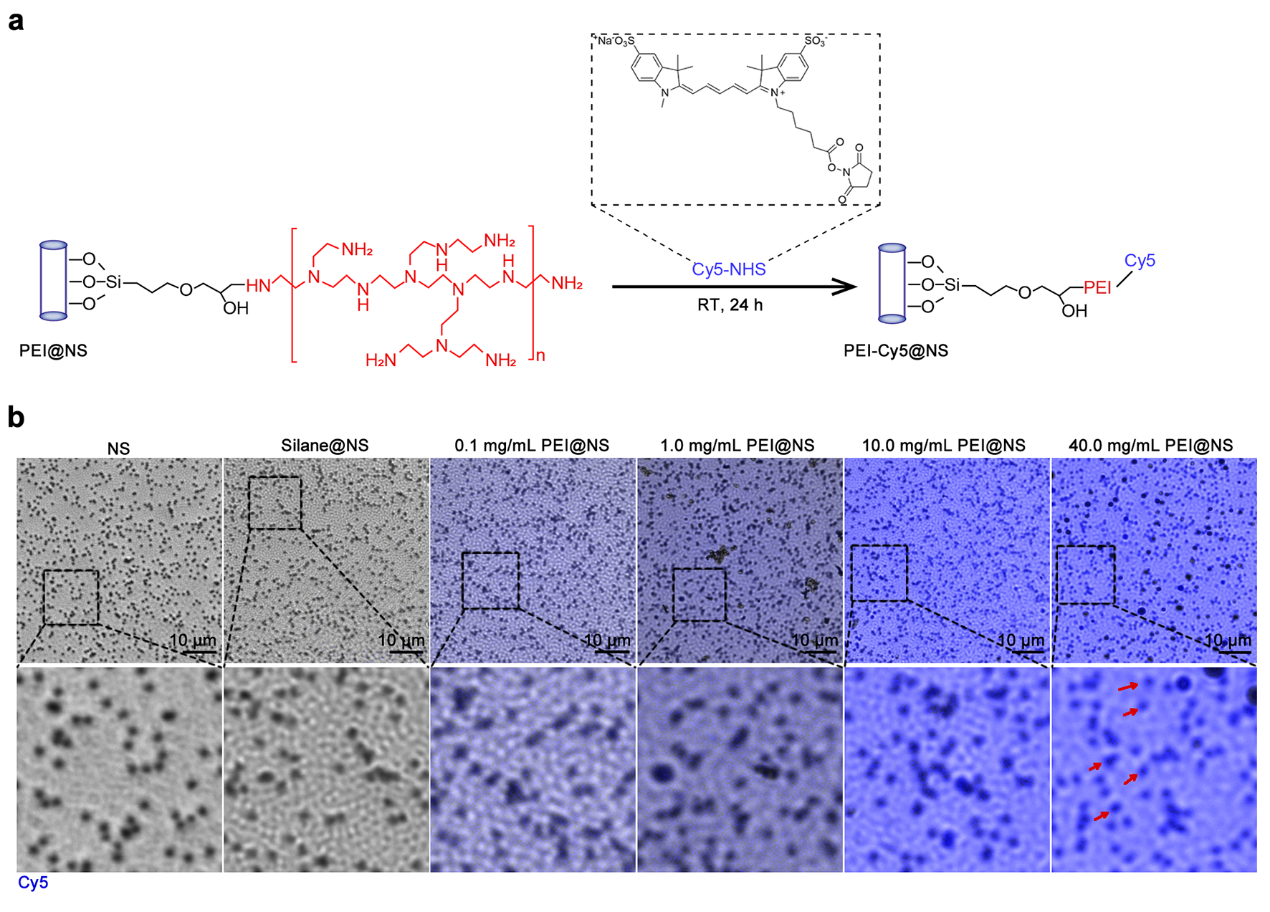


**Figure S4.** Validation of PEI modification onto NS. (a) Equations elucidating the reactions involved in the linking the fluorescent group, Cy5, with PEI@NS to form PEI-Cy5@NS. (b) Fluorescent microscopy images demonstrate the successful PEI-Cy5 (blue) modification onto the NS, with the enlarge view in the lower pannel. The red arrow represents the position of NS Scale bars are 10 μm.


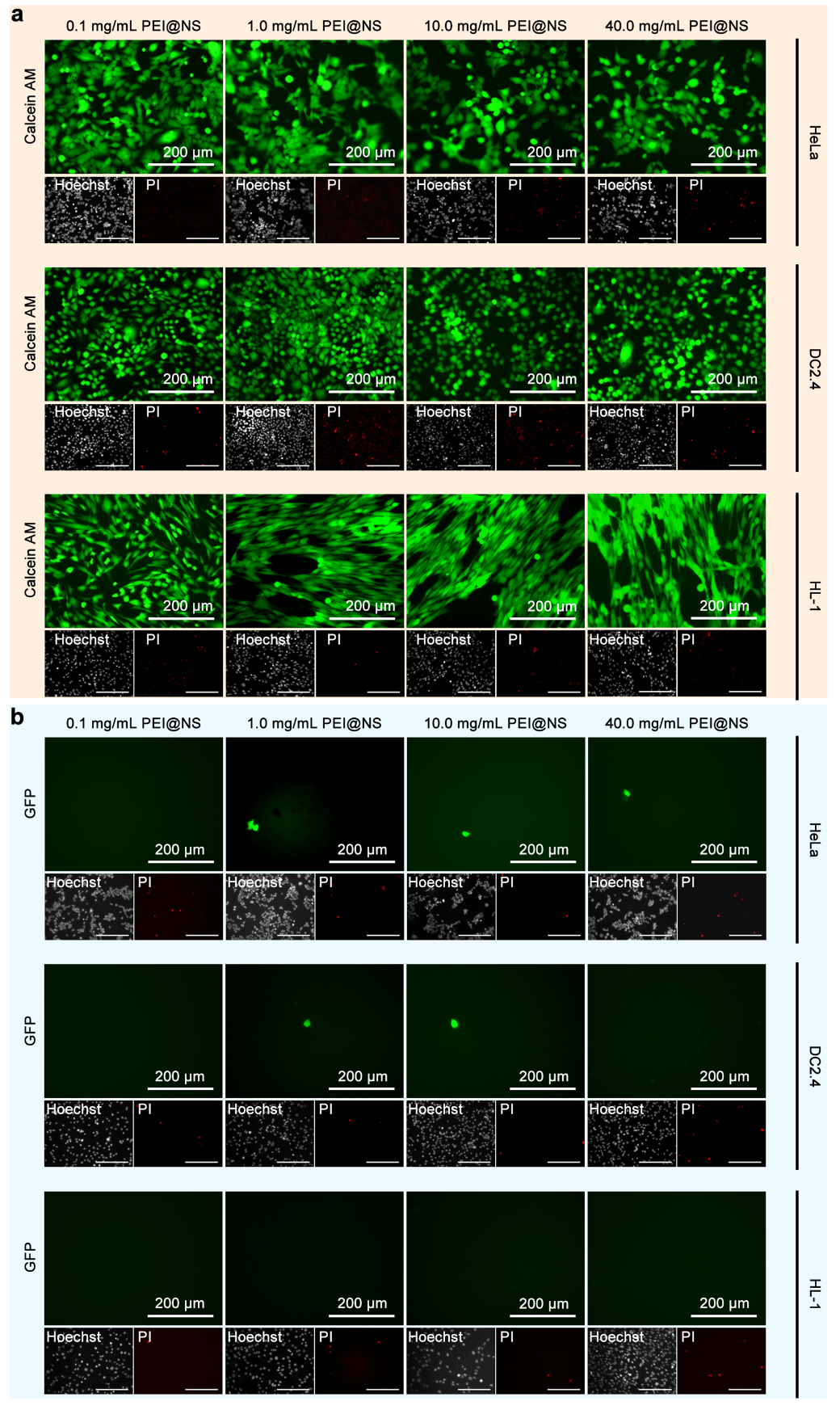


**Figure S5.** NS coupled with PEI modification for cellular DNA transfection. (a) Representative single-channel fluorescent microscopy images corresponding to Figure 3c depict the cellular condition after being cultured on PEI@NS. Calcein AM (green) highlights live cells, Hoechst (gray) stains nuclei, and PI (red) identifies dead cells. (b) Fluorescent microscopy images (corresponding to Figure 3e) demonstrate the transfection efficiency mediated by PEI@NS. GFP is represented in green, Hoechst in gray, and PI in red. Scale bars are 200 μm.


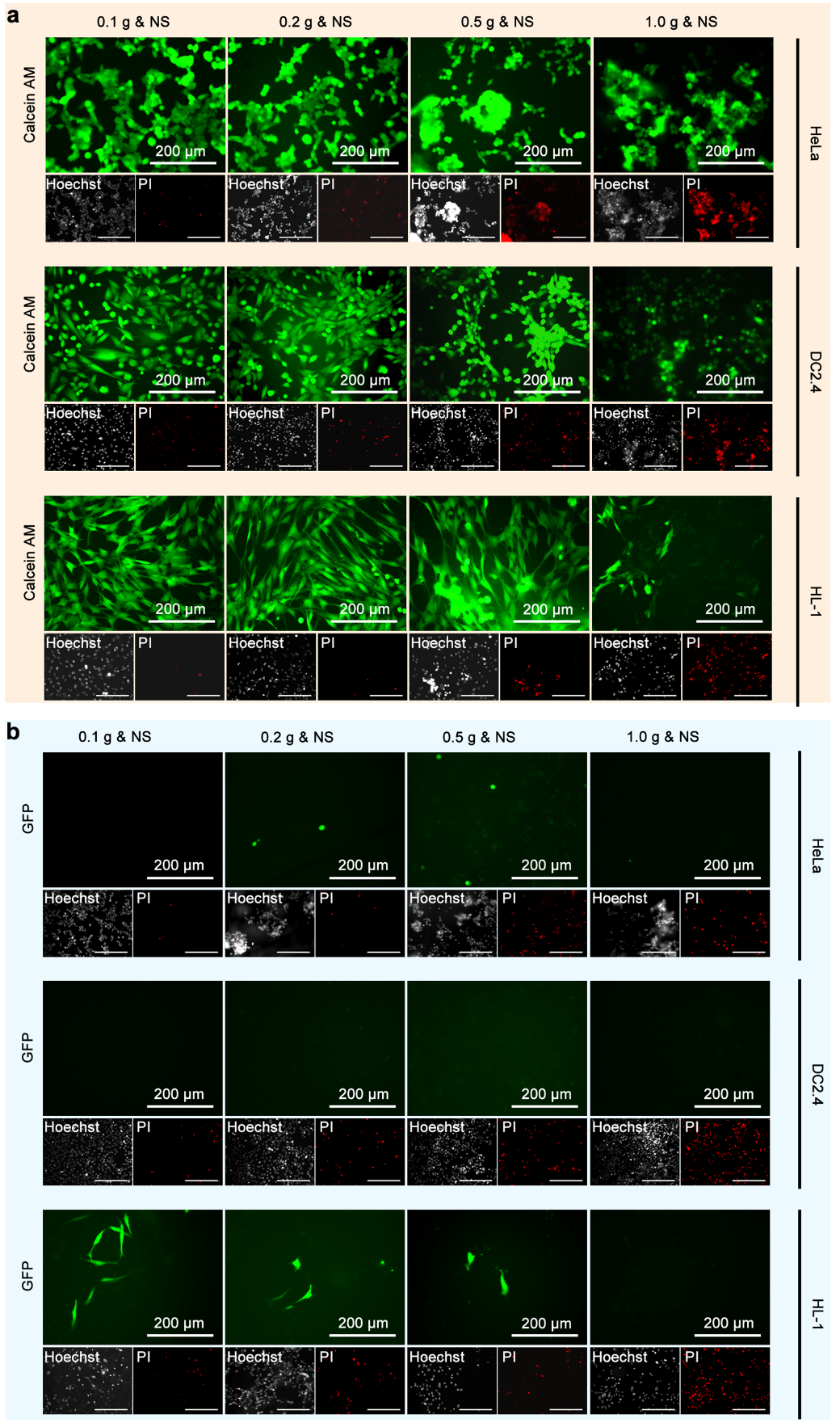


**Figure S6.** NS coupled with mechanical force applied to cellular DNA transfection. (a) Representative single-channel fluorescent microscopy images (corresponding to Figure 4c) depict cellular viability after exposure to external mechanical force. Live cells are highlighted in green using Calcein AM, nuclei are stained with Hoechst in gray, and dead cells are identified with PI in red. (b) Fluorescent microscopy images (corresponding to Figure 4e) illustrate the transfection efficiency mediated by NS coupled with mechanical force. GFP is represented in green, Hoechst gray, and PI in red. Scale bars are 200 μm.


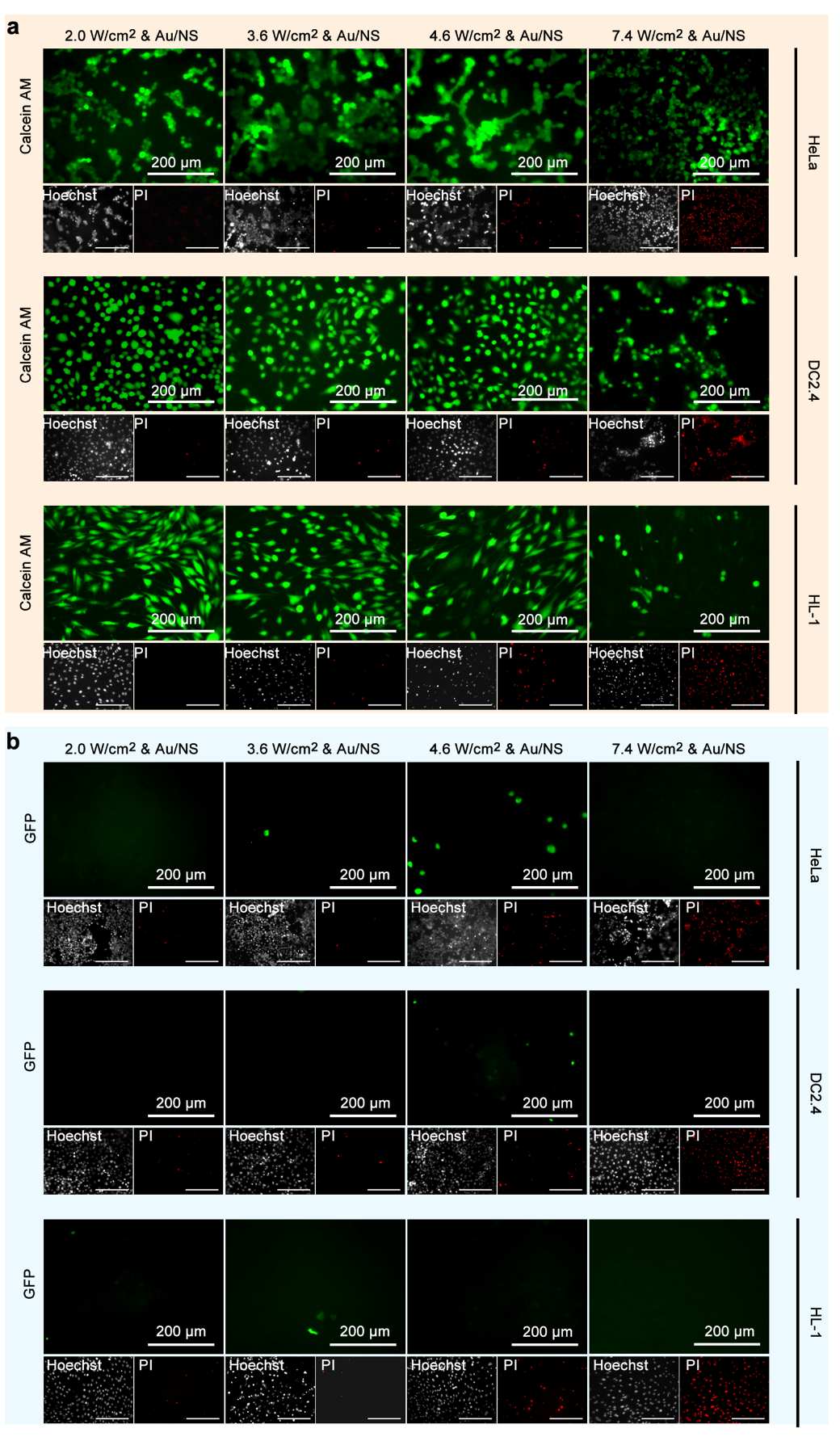


**Figure S7.** NS coupled with photothermal effect applied to cellular DNA transfection. (a) Representative single-channel fluorescent microscopy images (corresponding to Figure 5d) portray cellular viability following laser irradiation-induced photothermal effects of NS. Live cells are visualized in green using Calcein AM, nuclei are delineated in gray using Hoechst, and dead cells are identified in red with PI. (b) Fluorescent microscopy images (corresponding to Figure 5h) illustrate the transfection efficiency mediated by NS coupled with photothermal effect. Green fluorescence corresponds to GFP expression, Hoechst stains nuclei in gray, and PI highlights dead cells in red. Scale bars are 200 μm.


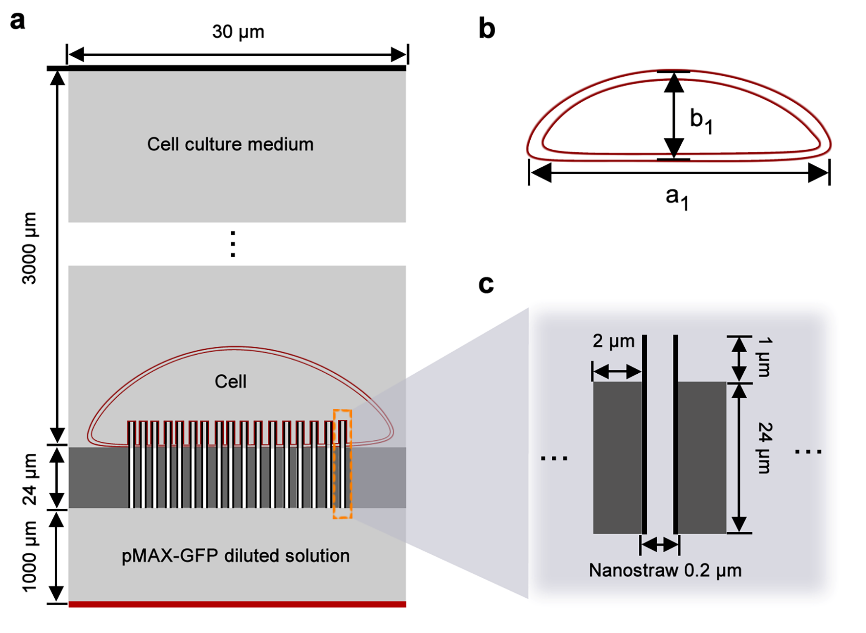


**Figure S8.** Schematic diagram with detailed dimensions of the 2D model for calculating the localized electrical field and transmembrane potential on the cell membrane. (a) The figure illustrates the entire 2D simulation model, with a conductive cell culture medium represented by grey rectangles above and below. The upper cell culture medium is partially shown due to size limitations. The grounding and given electric potential (V_0_) are indicated by the bold black line at the top and the bold red line at the bottom, respectively. (b) The semi-elliptical shape of the cell in the middle is defined by the "a1" semi-axis and the "b1" semi-axis dimensions, as provided in Supplementary Table 1. (c) The enlarged view of the TPM (shown in darker grey) housing NS (shown in black line) in the 2D model.

**Supplementary Table 1. Parameters of the cell shell in the 2D simulation model**

|  | a_1_ (μm) | b_1_ (μm) |
| --- | --- | --- |
| Outer membrane | 5 | 2.5 |
| Inner membrane | 4.994 | 2.488 |


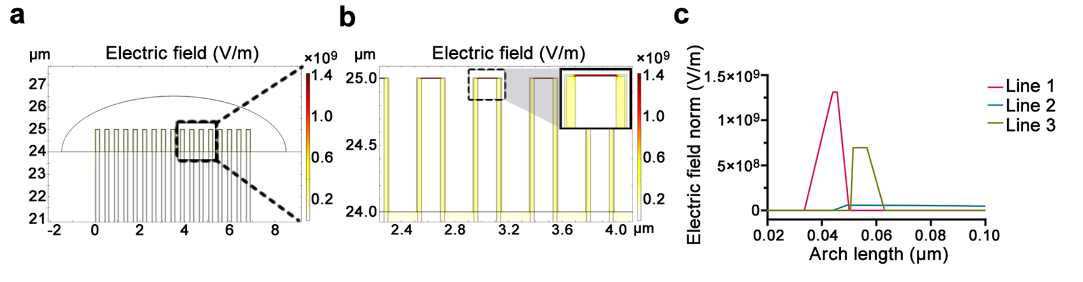


**Figure S9.** COMSOL simulation visually represents the electric field distribution at the cell-NS interface. (a, b) Display the accumulated norm intensity of the electric field at the interface between the nanosponge (NS) and the cell membrane. (c) Electric field profiles across the cell membrane along the intercept lines depicted in Figure 6g.


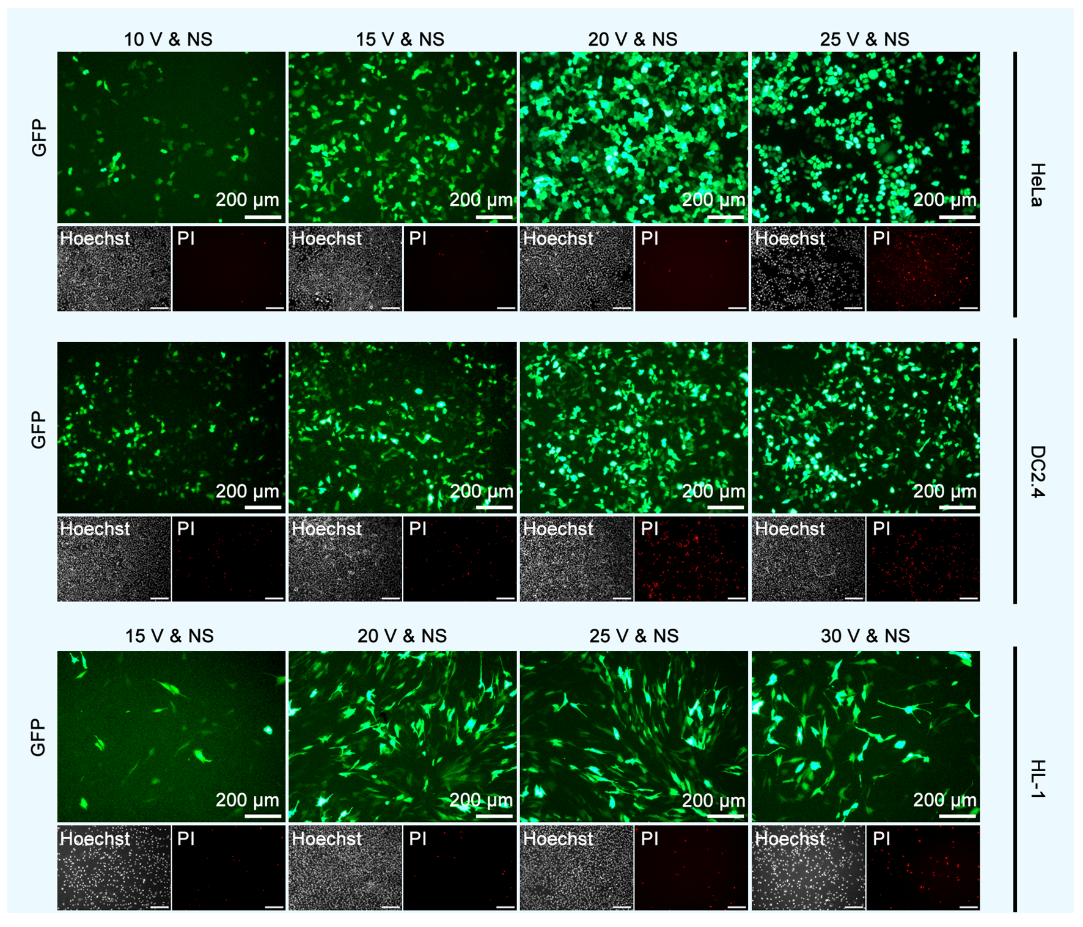


**Figure S10.** NS coupled with electric field for cellular DNA transfection. Fluorescent microscopy images (corresponding to Figure 6h) illustrate the transfection efficiency mediated by NS coupled with electric field. Green fluorescence corresponds to GFP expression, nuclei are stained in gray with Hoechst, and dead cells are highlighted in red with PI. Scale bars are 200 μm.
